# Supplementary material for: Identification of the regulatory elements and protein substrates of lysine acetoacetylation
Source: eLife. 2026 May 14;14:RP104123. doi: 10.7554/eLife.104123 (PMC13175576; doi:10.7554/eLife.104123)
Supplement: Figure 1—source data 1. [file elife-104123-fig1-data1.pdf]

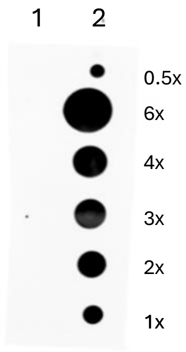

Figure 1, Source Data 1. Original membrane corresponding to Figure 1, panel B. Different dot sizes represent varying loading amounts of the H2BK15acac peptide, either without $NaBH_4$ reduction (lane 1) or with $NaBH_4$ reduction (lane 2), as detected by blotting with anti-Kbhb antibody.
