# Supplementary figures and images for: Identification of the regulatory elements and protein substrates of lysine acetoacetylation
Source: eLife. 2026 May 14;14:RP104123. doi: 10.7554/eLife.104123 (PMC13175576; doi:10.7554/eLife.104123)

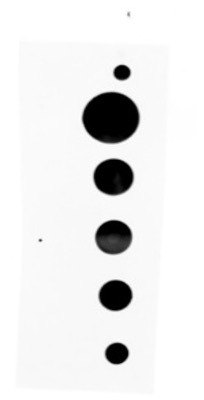

Supplement: Figure 1—source data 2. [file elife-104123-fig1-data2.zip › Figure 1-Source Data 2/Figure 1B-Dot blot results.jpg]

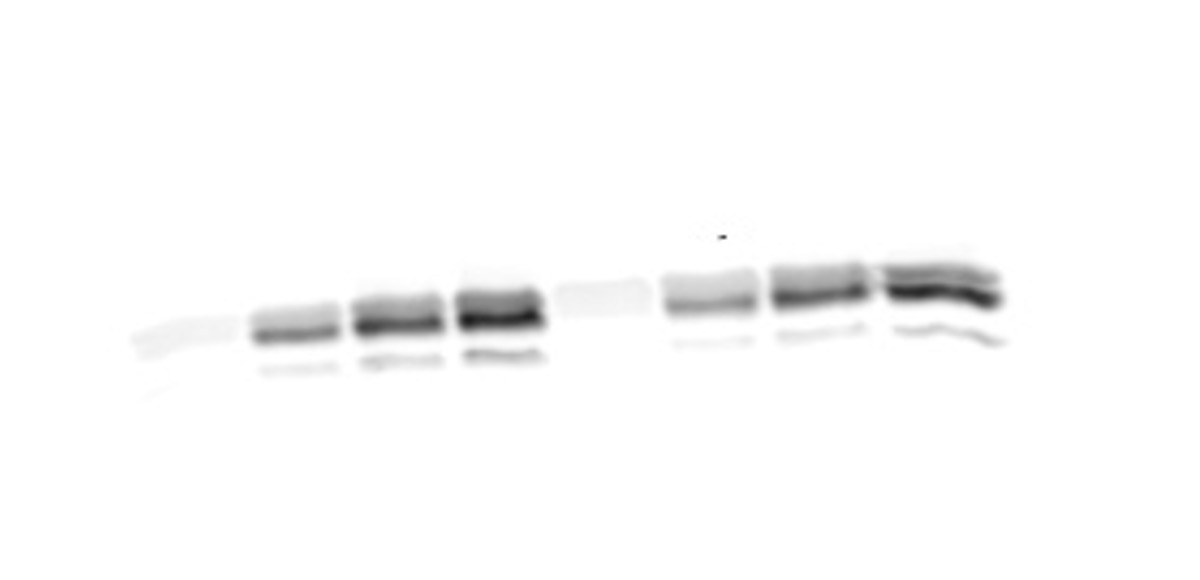

Supplement: Figure 2—source data 2. [file elife-104123-fig2-data2.zip › Figure 2-Source Data 2/Figure 2C-Kbhb western blot.jpg]

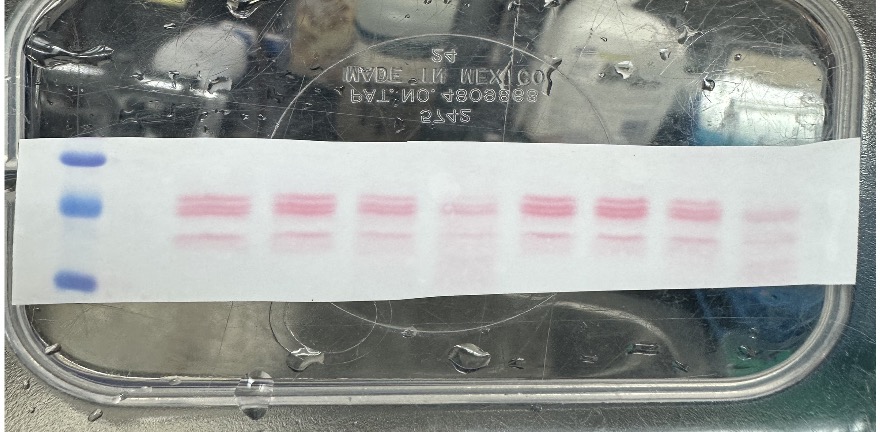

Supplement: Figure 2—source data 2. [file elife-104123-fig2-data2.zip › Figure 2-Source Data 2/Figure 2E-Ponceu S.jpg]

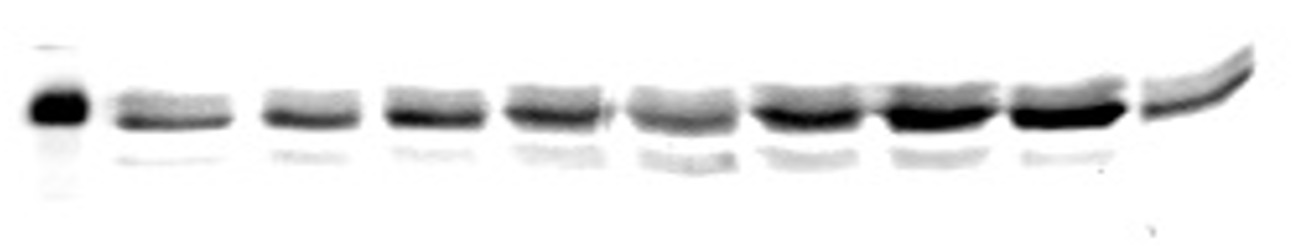

Supplement: Figure 2—source data 2. [file elife-104123-fig2-data2.zip › Figure 2-Source Data 2/Figure 2A-Kbhb western blot.jpg]

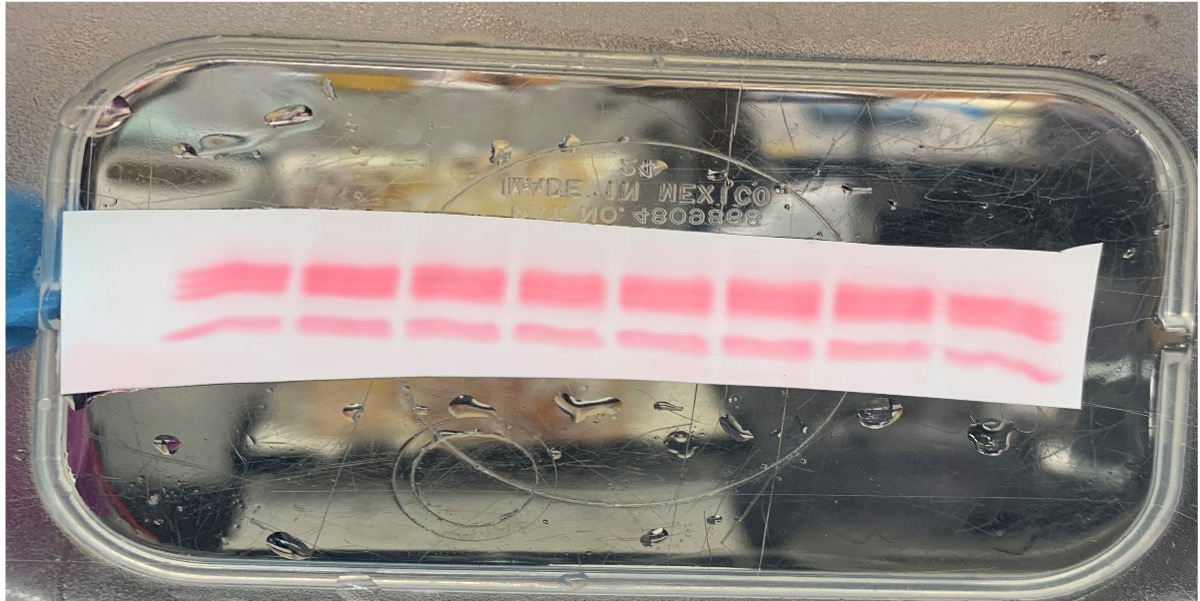

Supplement: Figure 2—source data 2. [file elife-104123-fig2-data2.zip › Figure 2-Source Data 2/Figure 2C-Ponceu S.jpg]

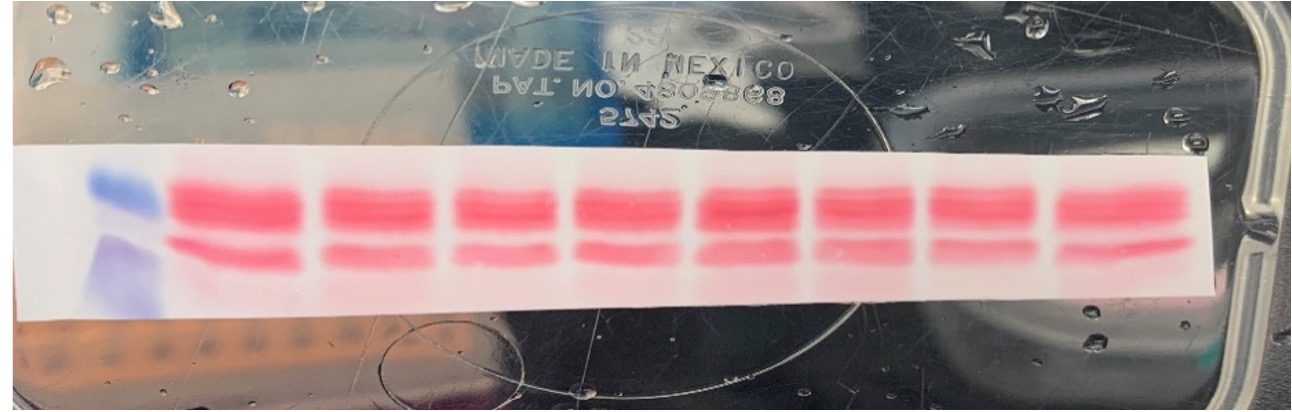

Supplement: Figure 2—source data 2. [file elife-104123-fig2-data2.zip › Figure 2-Source Data 2/Figure 2D-Ponceu S.jpg]

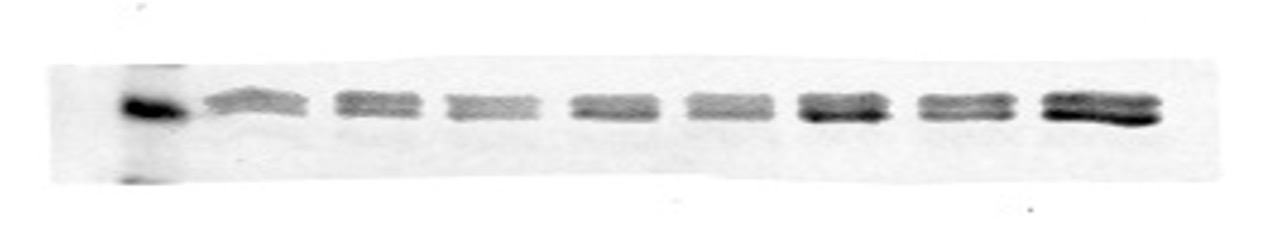

Supplement: Figure 2—source data 2. [file elife-104123-fig2-data2.zip › Figure 2-Source Data 2/Figure 2B-Kbhb western blot (last 4 lanes).jpg]

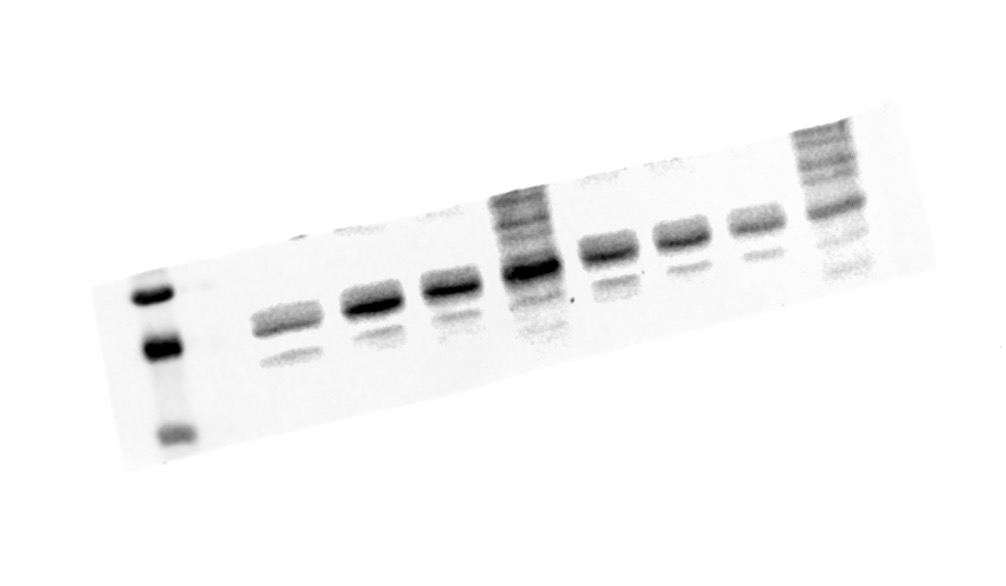

Supplement: Figure 2—source data 2. [file elife-104123-fig2-data2.zip › Figure 2-Source Data 2/Figure 2E-Kbhb western blot.jpg]

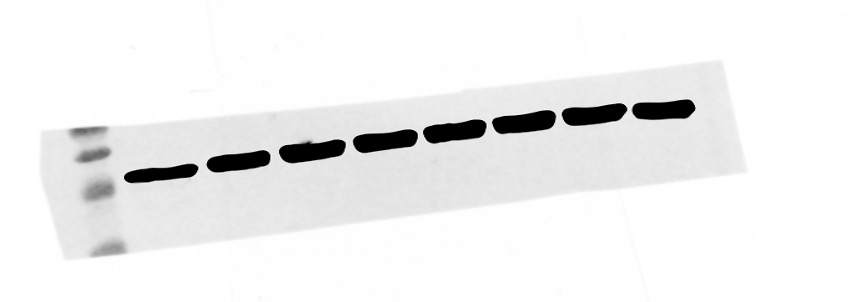

Supplement: Figure 2—source data 2. [file elife-104123-fig2-data2.zip › Figure 2-Source Data 2/Figure 2F-H3 loading.jpg]

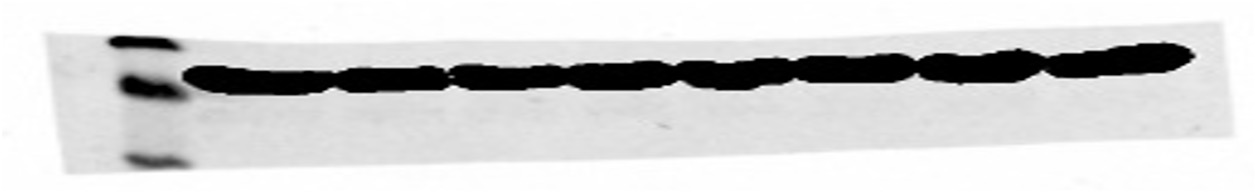

Supplement: Figure 2—source data 2. [file elife-104123-fig2-data2.zip › Figure 2-Source Data 2/Figure 2A-H3 loading.jpg]

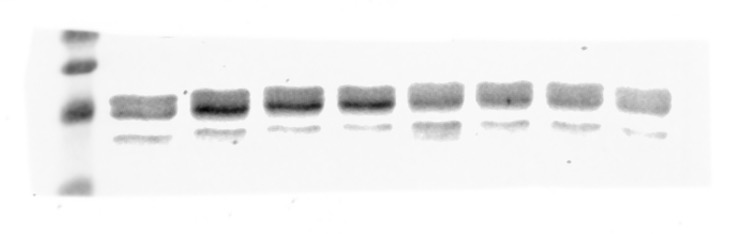

Supplement: Figure 2—source data 2. [file elife-104123-fig2-data2.zip › Figure 2-Source Data 2/Figure 2F-Kbhb western blot.jpg]

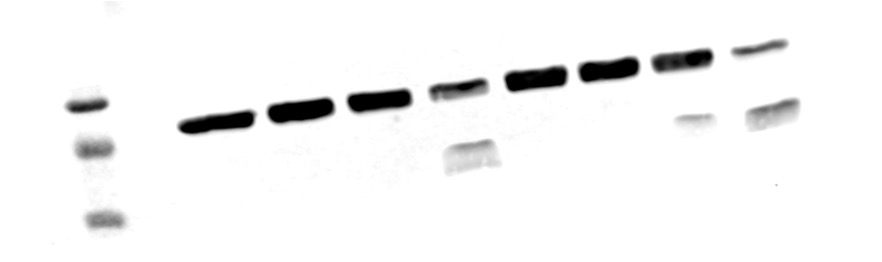

Supplement: Figure 2—source data 2. [file elife-104123-fig2-data2.zip › Figure 2-Source Data 2/Figure 2E-H3 loading.jpg]

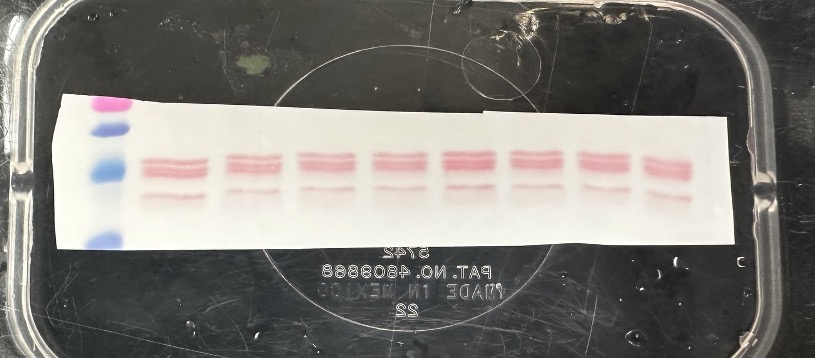

Supplement: Figure 2—source data 2. [file elife-104123-fig2-data2.zip › Figure 2-Source Data 2/Figure 2F-Ponceu S.jpg]

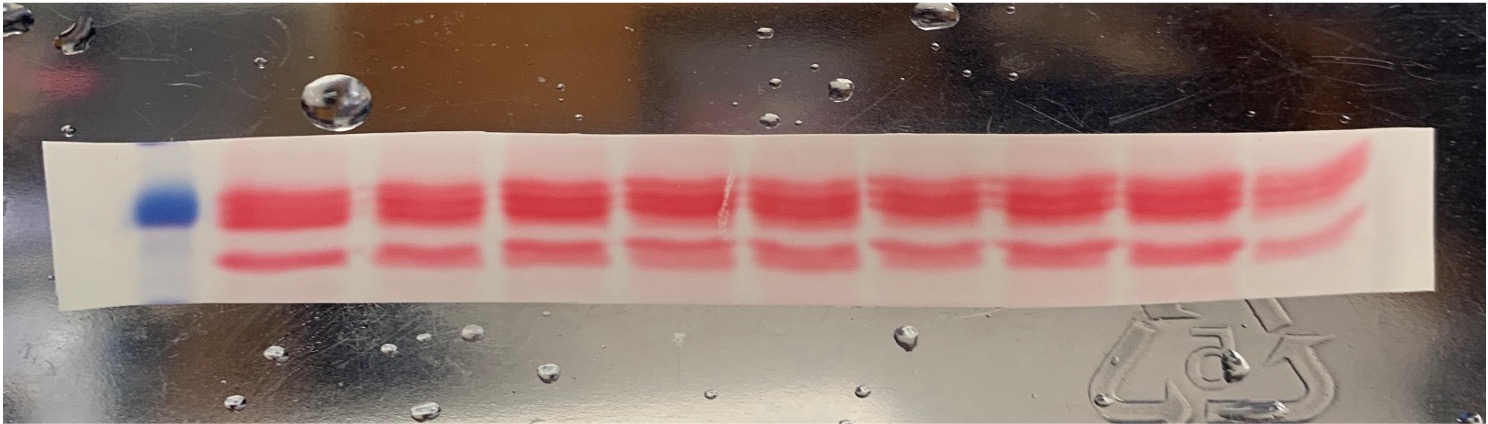

Supplement: Figure 2—source data 2. [file elife-104123-fig2-data2.zip › Figure 2-Source Data 2/Figure 2A-Ponceu S.jpg]

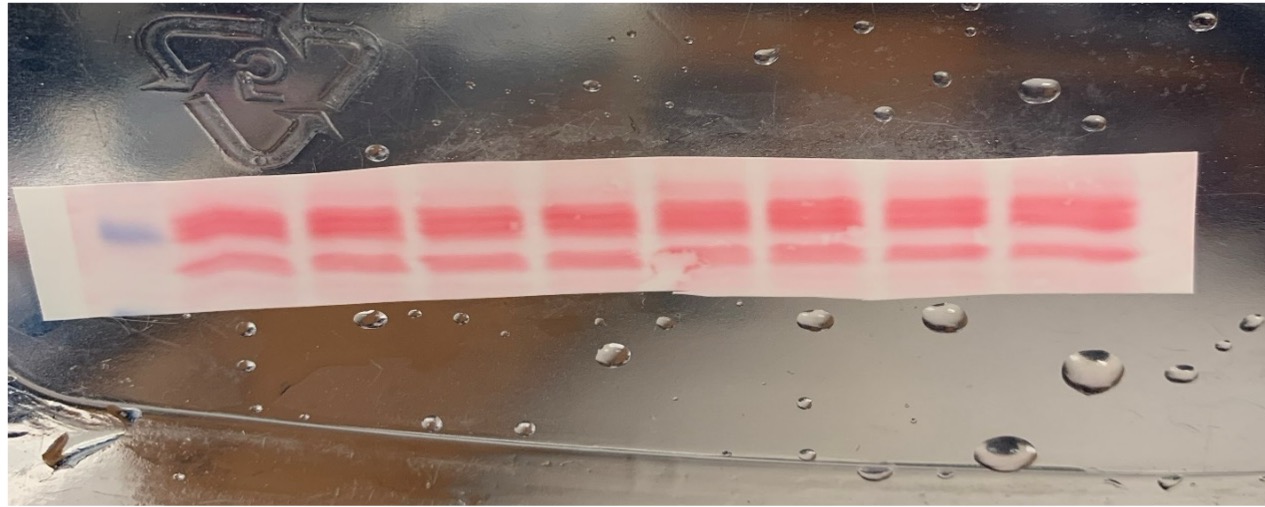

Supplement: Figure 2—source data 2. [file elife-104123-fig2-data2.zip › Figure 2-Source Data 2/Figure 2B-Ponceu S (last 4 lanes).jpg]

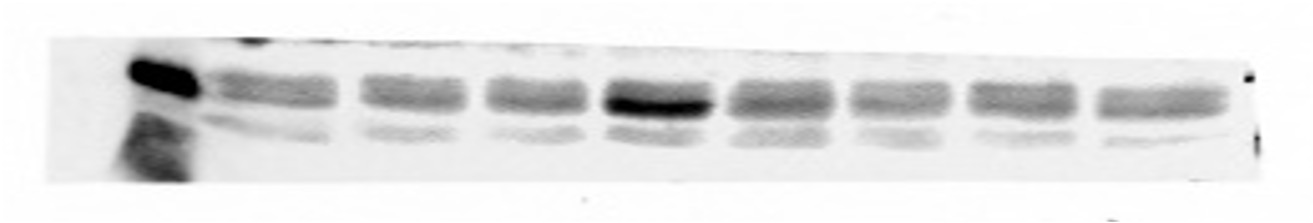

Supplement: Figure 2—source data 2. [file elife-104123-fig2-data2.zip › Figure 2-Source Data 2/Figure 2D-Kbhb western blot.jpg]

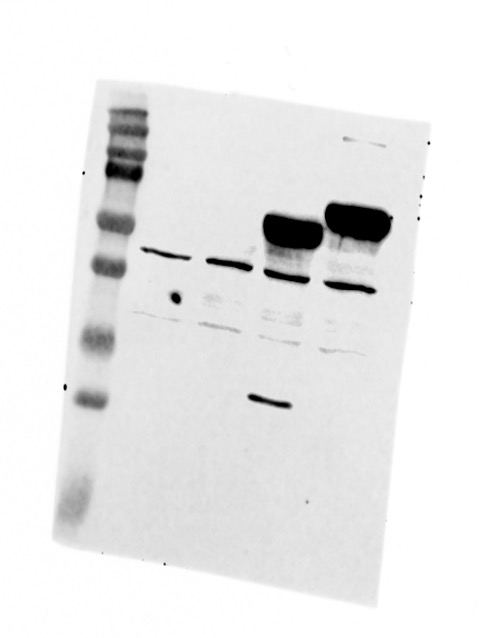

Supplement: Figure 2—figure supplement 1—source data 2. [file elife-104123-fig2-figsupp1-data2.zip › Figure 2-figure supplement 1-Source Data 2/Figure 2-figure supplement 1B-actin loading.jpg]

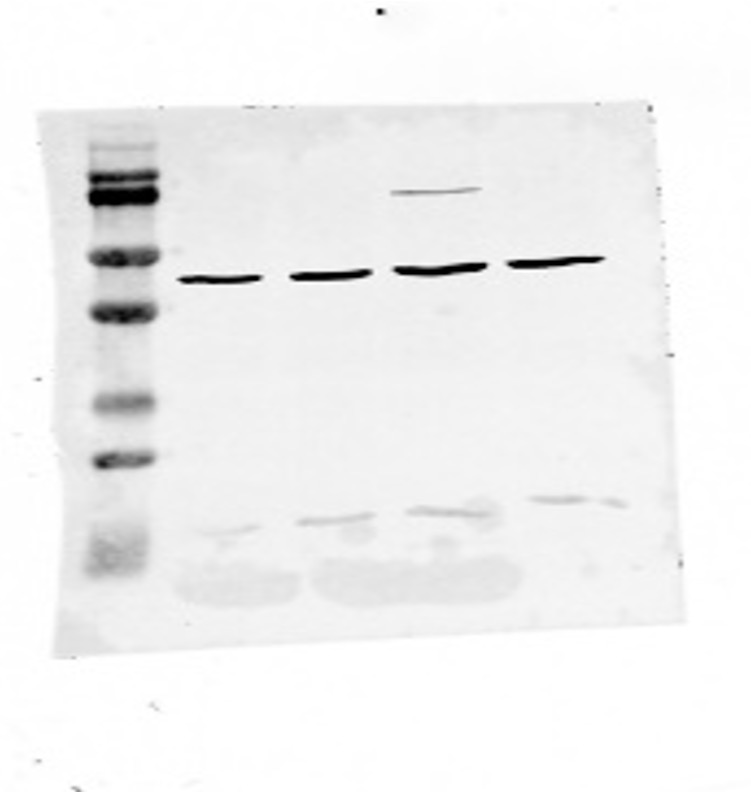

Supplement: Figure 2—figure supplement 1—source data 2. [file elife-104123-fig2-figsupp1-data2.zip › Figure 2-figure supplement 1-Source Data 2/Figure 2-figure supplement 1C-Actin loading.jpg]

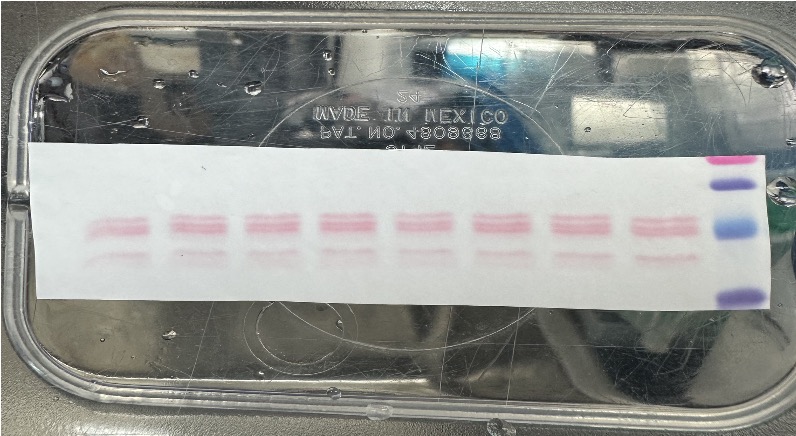

Supplement: Figure 2—figure supplement 1—source data 2. [file elife-104123-fig2-figsupp1-data2.zip › Figure 2-figure supplement 1-Source Data 2/Figure 2-figure supplement 1D-Ponceau S.jpg]

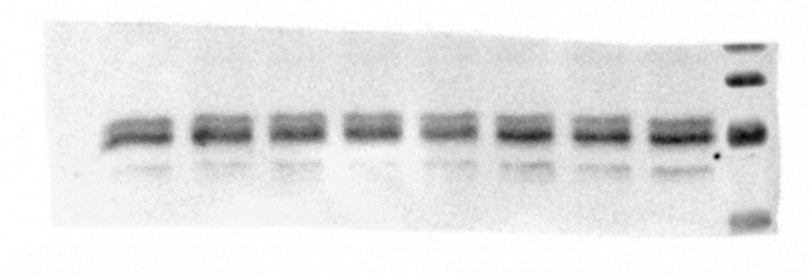

Supplement: Figure 2—figure supplement 1—source data 2. [file elife-104123-fig2-figsupp1-data2.zip › Figure 2-figure supplement 1-Source Data 2/Figure 2-figure supplement 1D-Kbhb western blot.jpg]

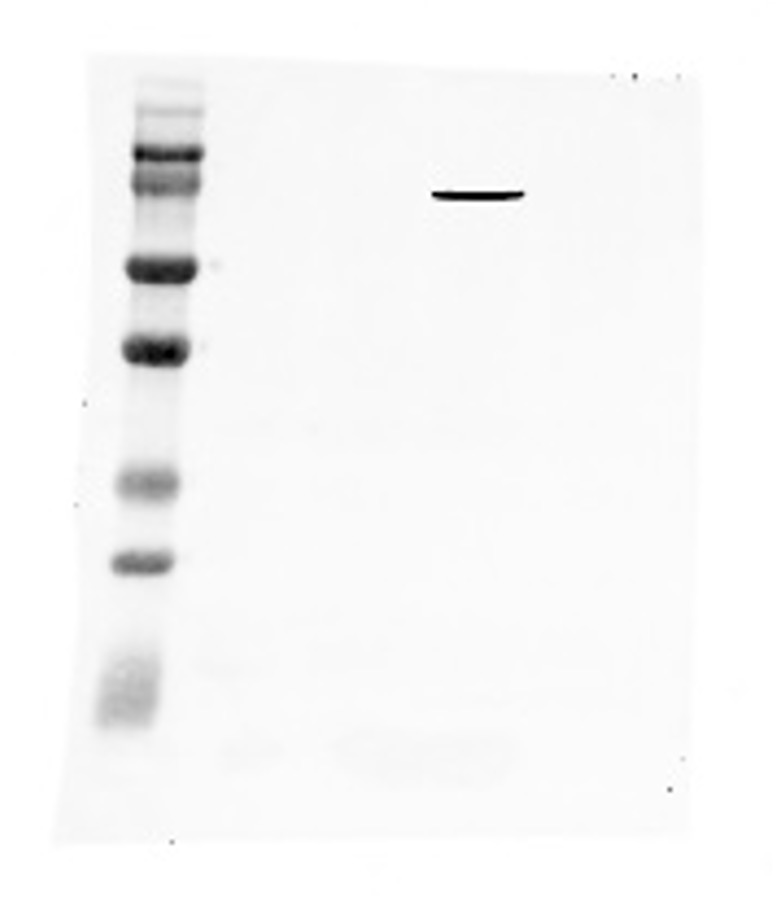

Supplement: Figure 2—figure supplement 1—source data 2. [file elife-104123-fig2-figsupp1-data2.zip › Figure 2-figure supplement 1-Source Data 2/Figure 2-figure supplement 1C-Anti-flag western blot.jpg]

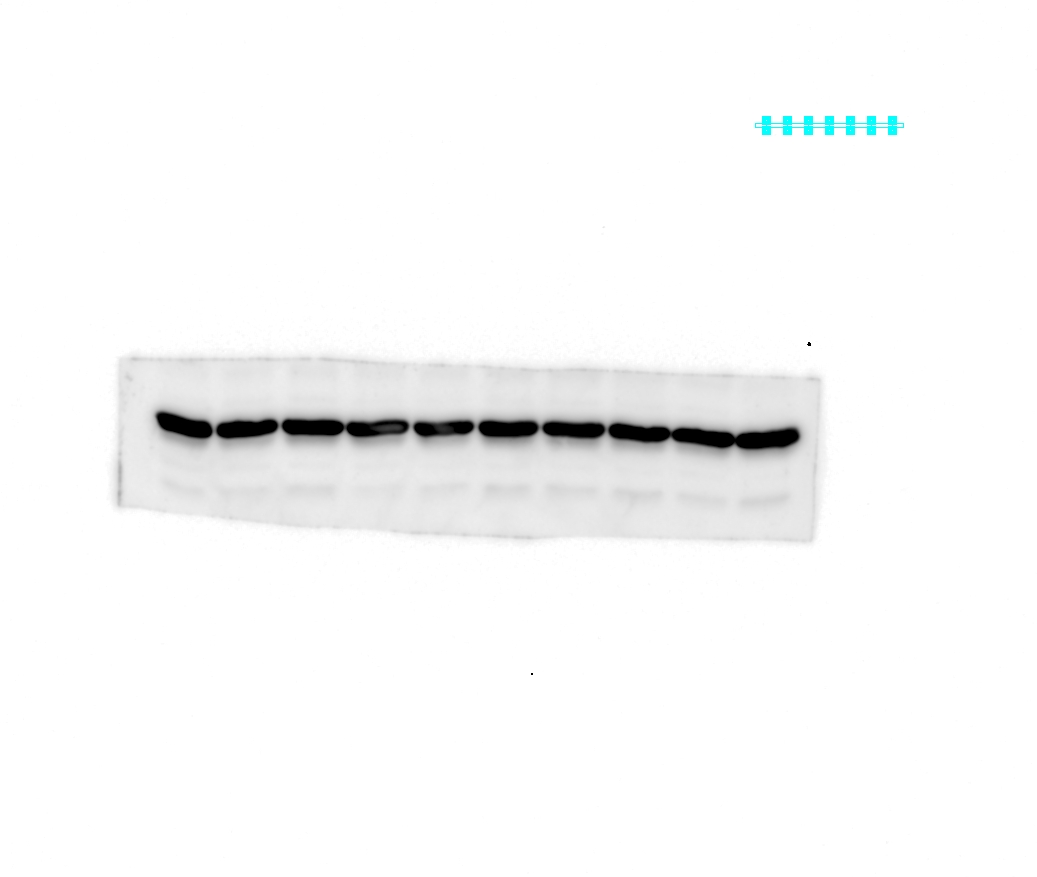

Supplement: Figure 2—figure supplement 1—source data 2. [file elife-104123-fig2-figsupp1-data2.zip › Figure 2-figure supplement 1-Source Data 2/Figure 2-figure supplement 1A-H3 loading.jpg]

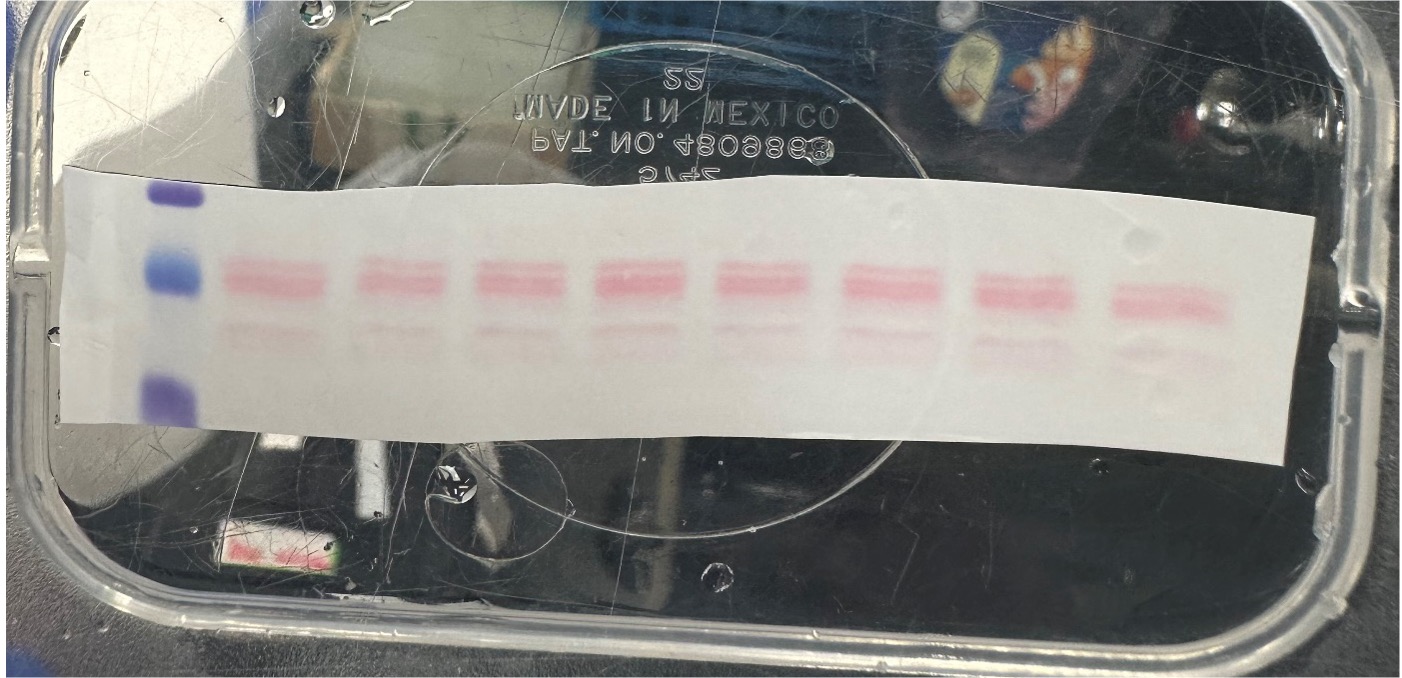

Supplement: Figure 2—figure supplement 1—source data 2. [file elife-104123-fig2-figsupp1-data2.zip › Figure 2-figure supplement 1-Source Data 2/Figure 2-figure supplement 1E-Ponceau S.jpg]

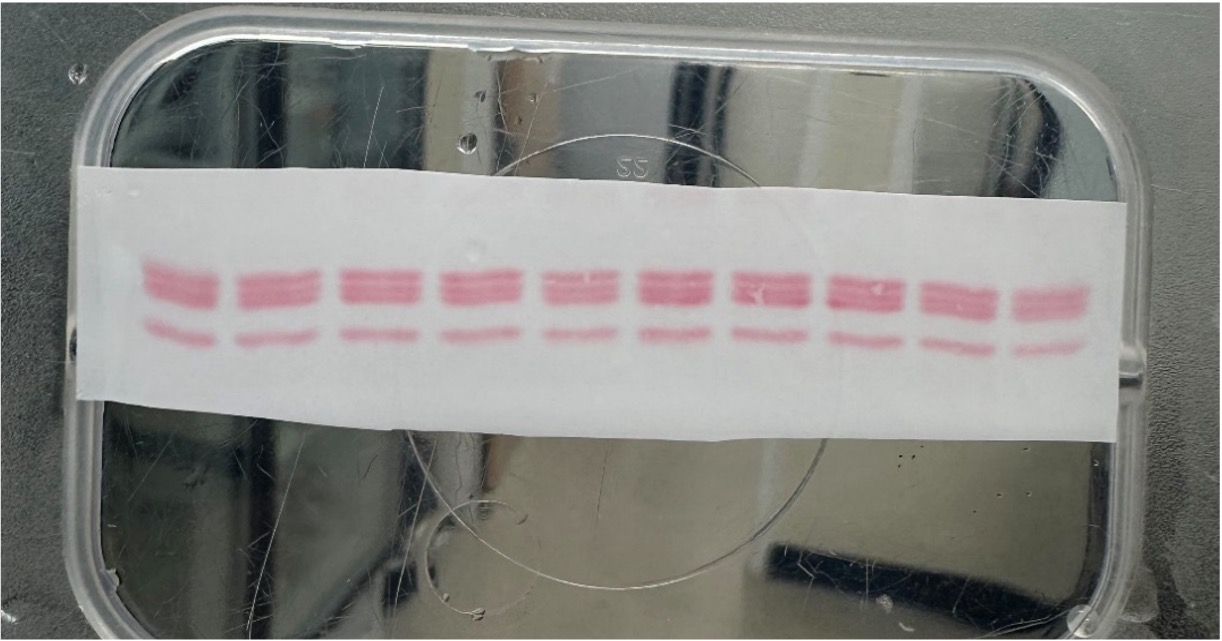

Supplement: Figure 2—figure supplement 1—source data 2. [file elife-104123-fig2-figsupp1-data2.zip › Figure 2-figure supplement 1-Source Data 2/Figure 2-figure supplement 1A-Ponceau S.jpg]

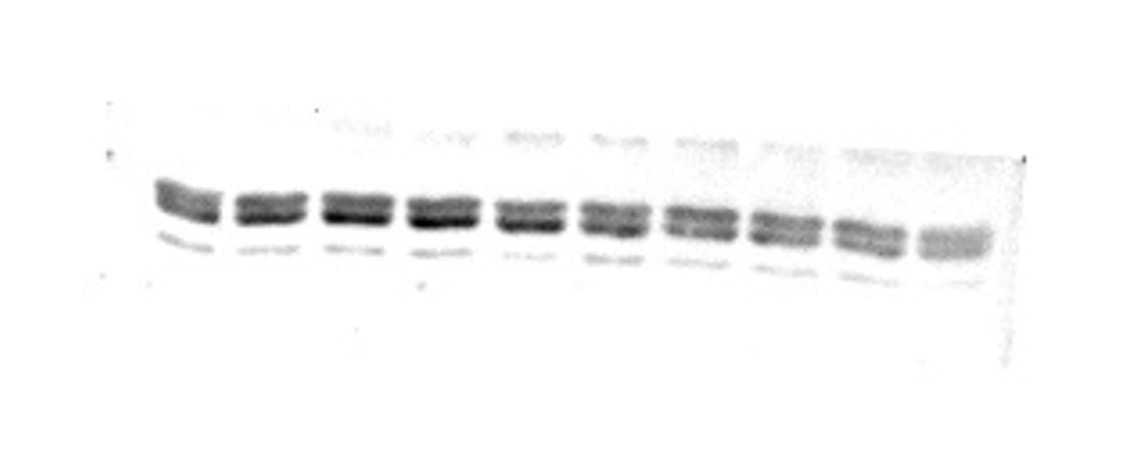

Supplement: Figure 2—figure supplement 1—source data 2. [file elife-104123-fig2-figsupp1-data2.zip › Figure 2-figure supplement 1-Source Data 2/Figure 2-figure supplement 1A-Kbhb western blot.jpg]

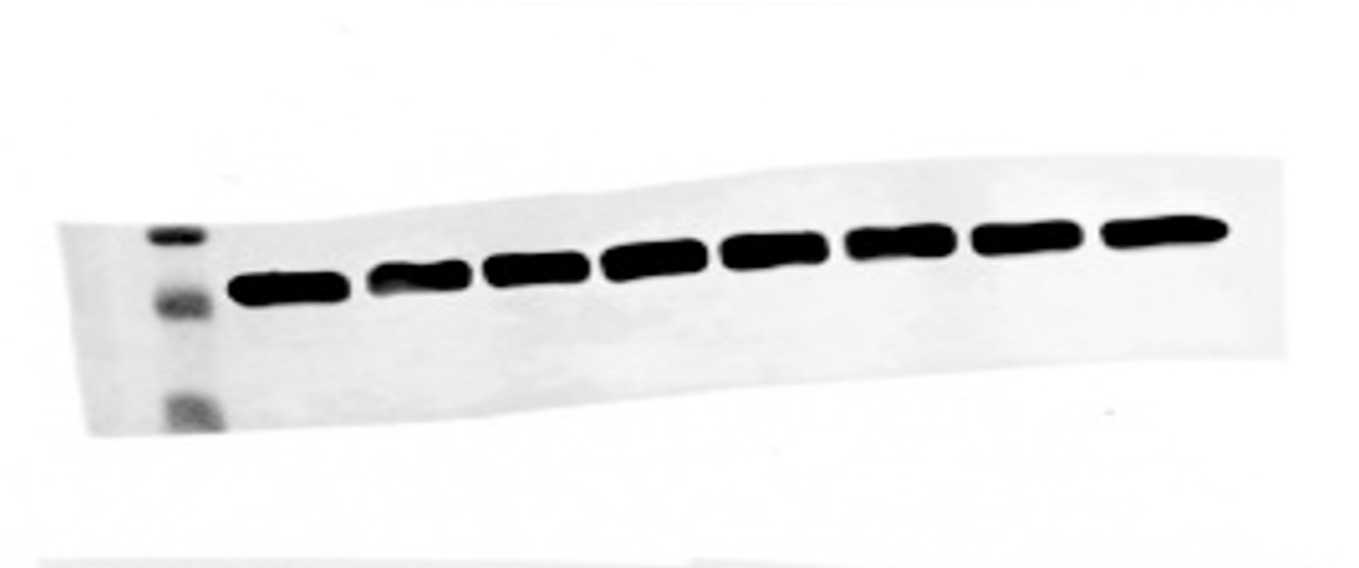

Supplement: Figure 2—figure supplement 1—source data 2. [file elife-104123-fig2-figsupp1-data2.zip › Figure 2-figure supplement 1-Source Data 2/Figure 2-figure supplement 1E-H3 loading.jpg]

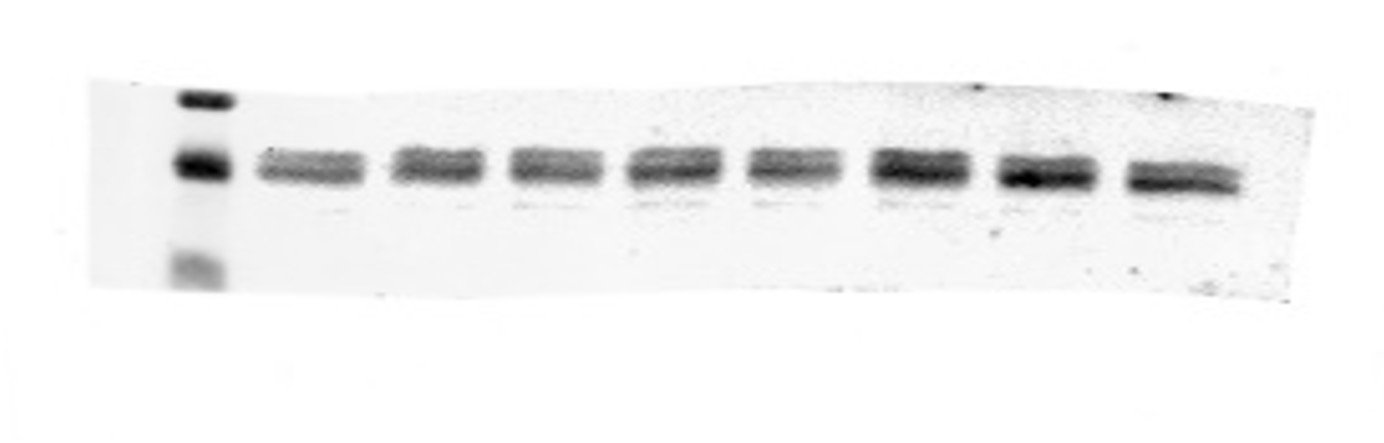

Supplement: Figure 2—figure supplement 1—source data 2. [file elife-104123-fig2-figsupp1-data2.zip › Figure 2-figure supplement 1-Source Data 2/Figure 2-figure supplement 1E-Kbhb western blot.jpg]

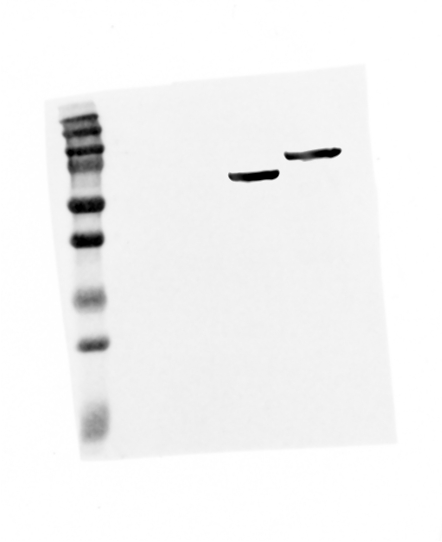

Supplement: Figure 2—figure supplement 1—source data 2. [file elife-104123-fig2-figsupp1-data2.zip › Figure 2-figure supplement 1-Source Data 2/Figure 2-figure supplement 1B-Anti-flag western blot.jpg]

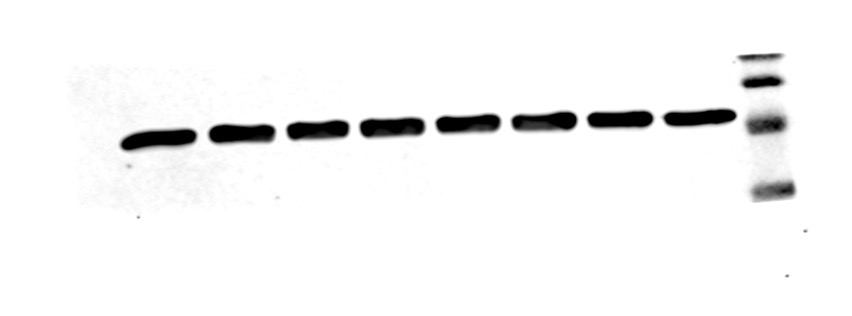

Supplement: Figure 2—figure supplement 1—source data 2. [file elife-104123-fig2-figsupp1-data2.zip › Figure 2-figure supplement 1-Source Data 2/Figure 2-figure supplement 1D-H3 loading.jpg]

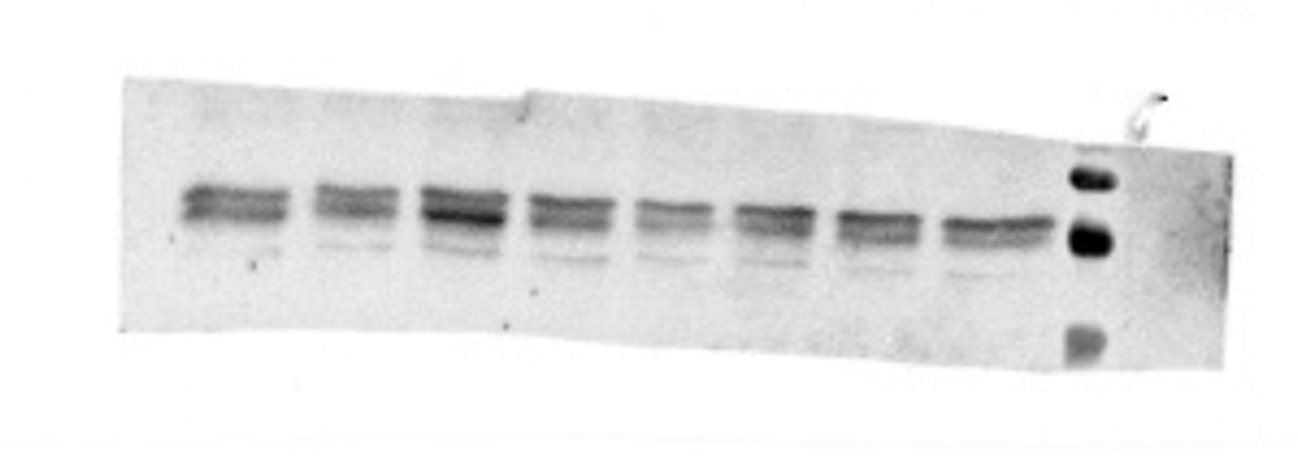

Supplement: Figure 3—source data 2. [file elife-104123-fig3-data2.zip › Figure 3-Source Data 2/Figure 3E-Kbhb western blot.jpg]

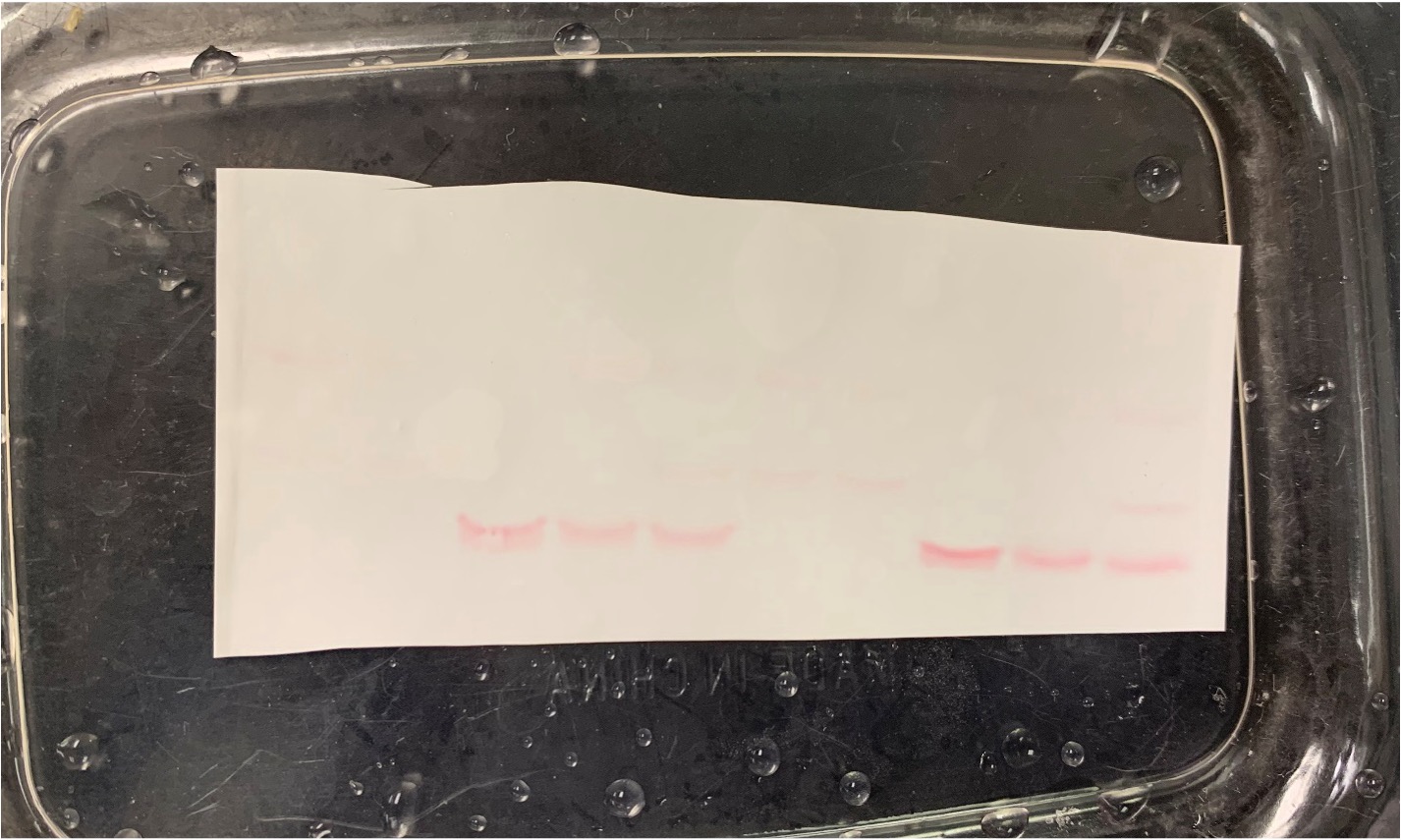

Supplement: Figure 3—source data 2. [file elife-104123-fig3-data2.zip › Figure 3-Source Data 2/Figure 3B-Ponceau S.jpg]

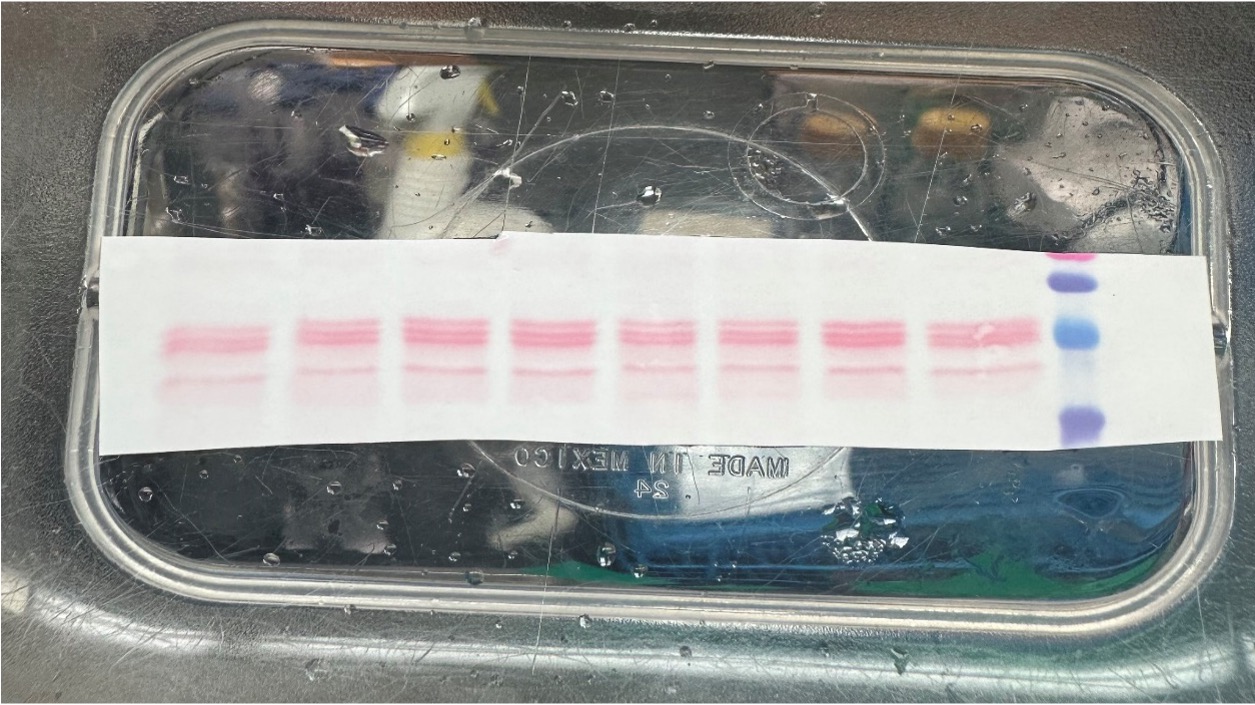

Supplement: Figure 3—source data 2. [file elife-104123-fig3-data2.zip › Figure 3-Source Data 2/Figure 3E-Ponceau S.jpg]

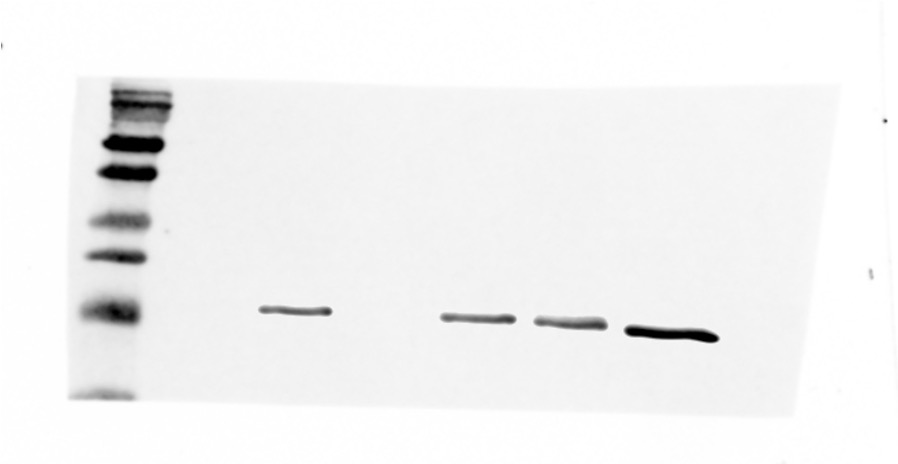

Supplement: Figure 3—source data 2. [file elife-104123-fig3-data2.zip › Figure 3-Source Data 2/Figure 3C-Kac western blot.jpg]

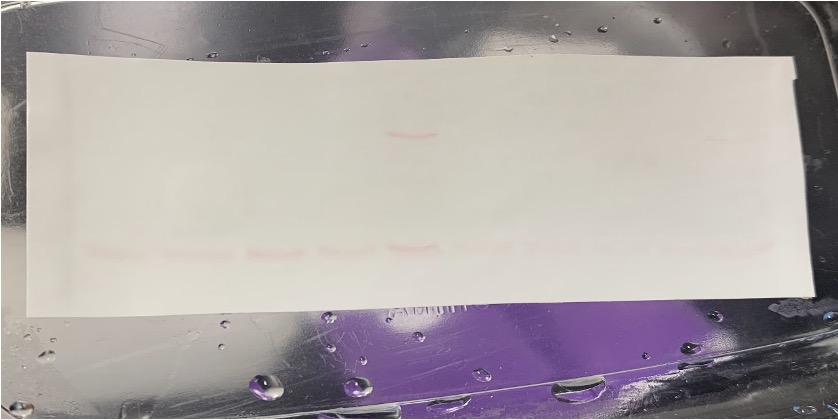

Supplement: Figure 3—source data 2. [file elife-104123-fig3-data2.zip › Figure 3-Source Data 2/Figure 3A-Ponceau S-left panel.jpg]

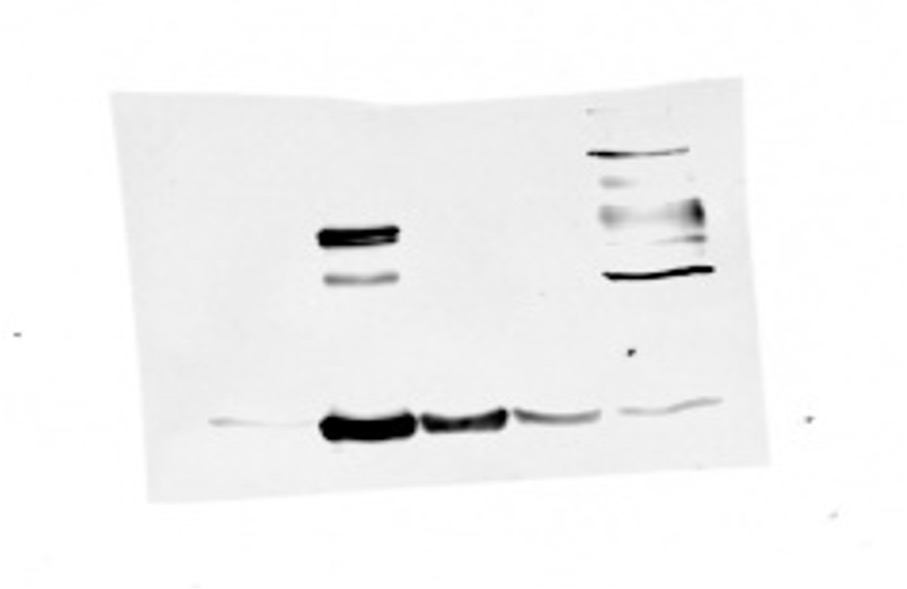

Supplement: Figure 3—source data 2. [file elife-104123-fig3-data2.zip › Figure 3-Source Data 2/Figure 3A-Kac western blot-right panel.jpg]

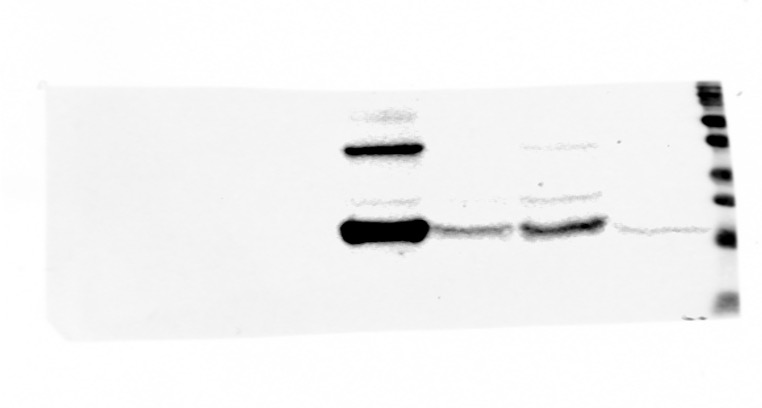

Supplement: Figure 3—source data 2. [file elife-104123-fig3-data2.zip › Figure 3-Source Data 2/Figure 3C-Kbhb western blot.jpg]

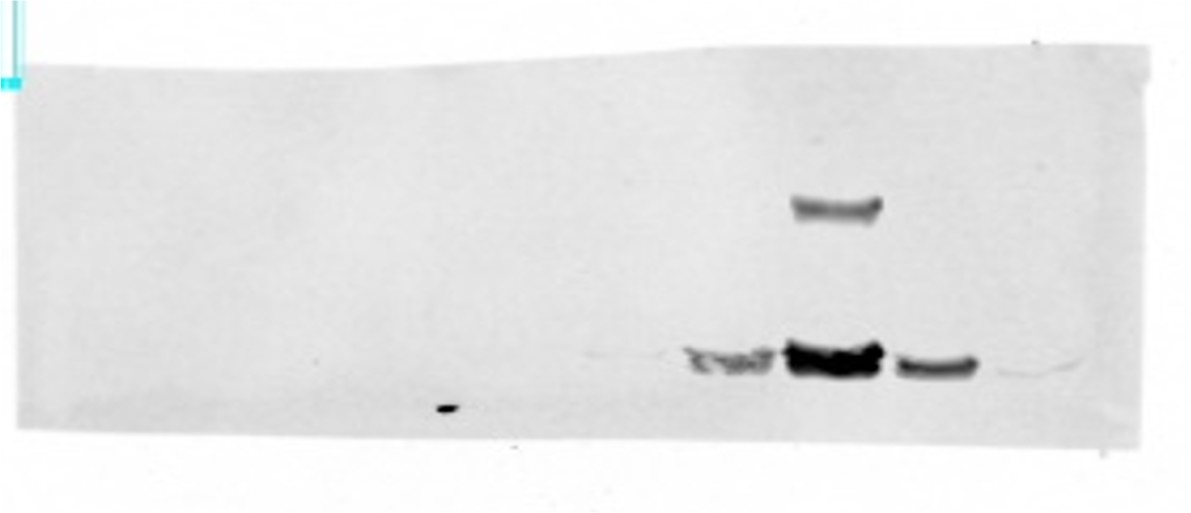

Supplement: Figure 3—source data 2. [file elife-104123-fig3-data2.zip › Figure 3-Source Data 2/Figure 3A-Kbhb western blot-left panel.jpg]

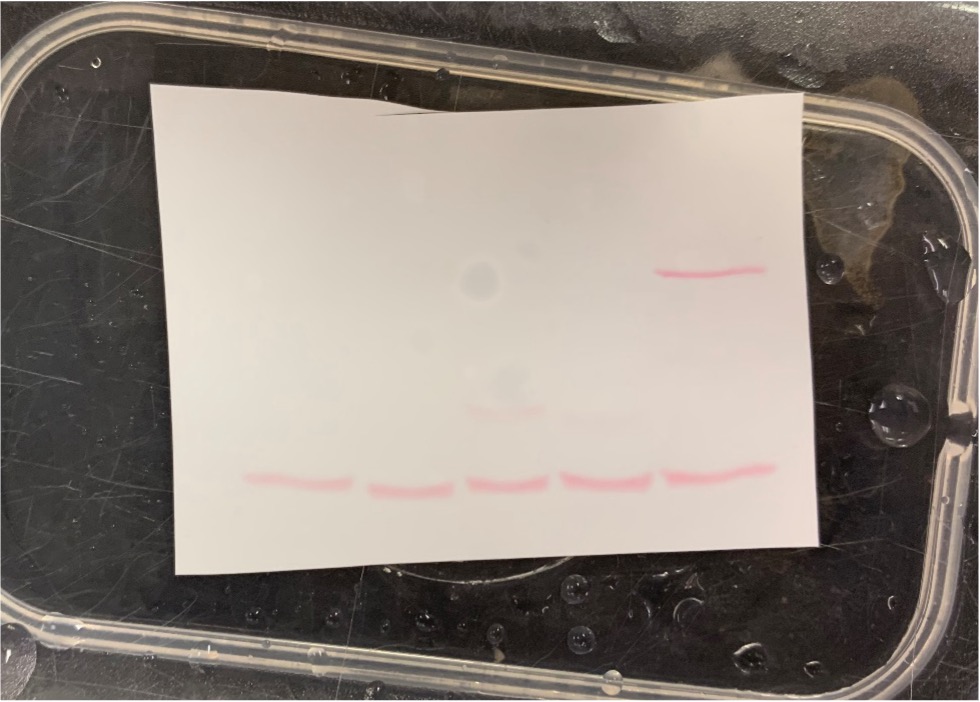

Supplement: Figure 3—source data 2. [file elife-104123-fig3-data2.zip › Figure 3-Source Data 2/Figure 3A-Ponceau S-right panel.jpg]

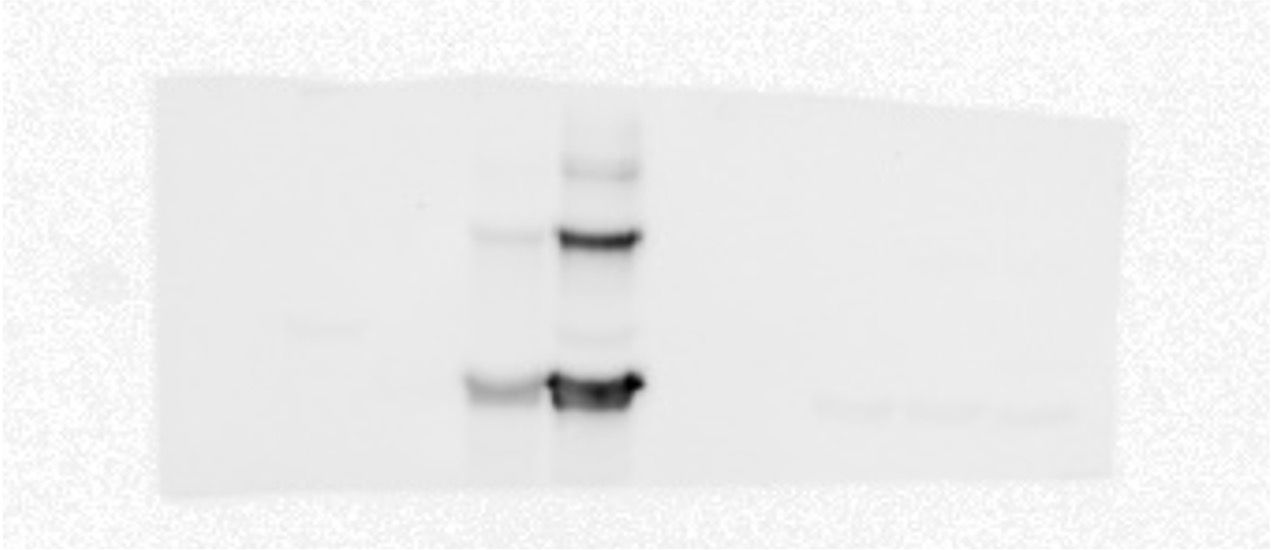

Supplement: Figure 3—source data 2. [file elife-104123-fig3-data2.zip › Figure 3-Source Data 2/Figure 3B-Kbhb western blot.jpg]

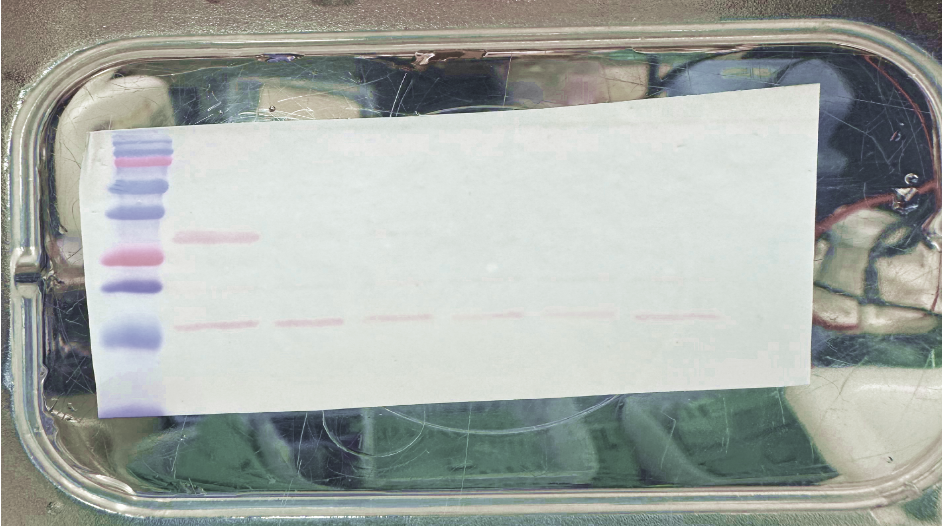

Supplement: Figure 3—source data 2. [file elife-104123-fig3-data2.zip › Figure 3-Source Data 2/Figure 3C-Ponceau S.jpg]

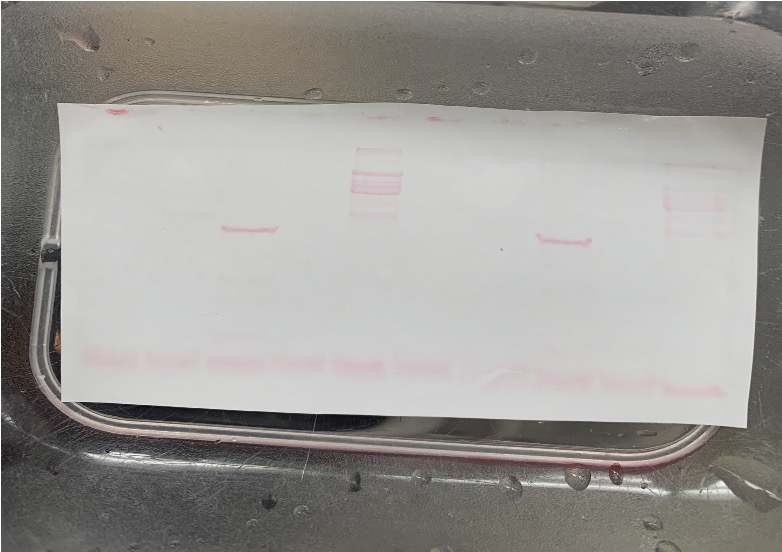

Supplement: Figure 3—figure supplement 1—source data 2. [file elife-104123-fig3-figsupp1-data2.zip › Figure 3-figure supplement 1-Source Data 2/Figure 3-figure supplement 1D-Ponceau S.jpg]

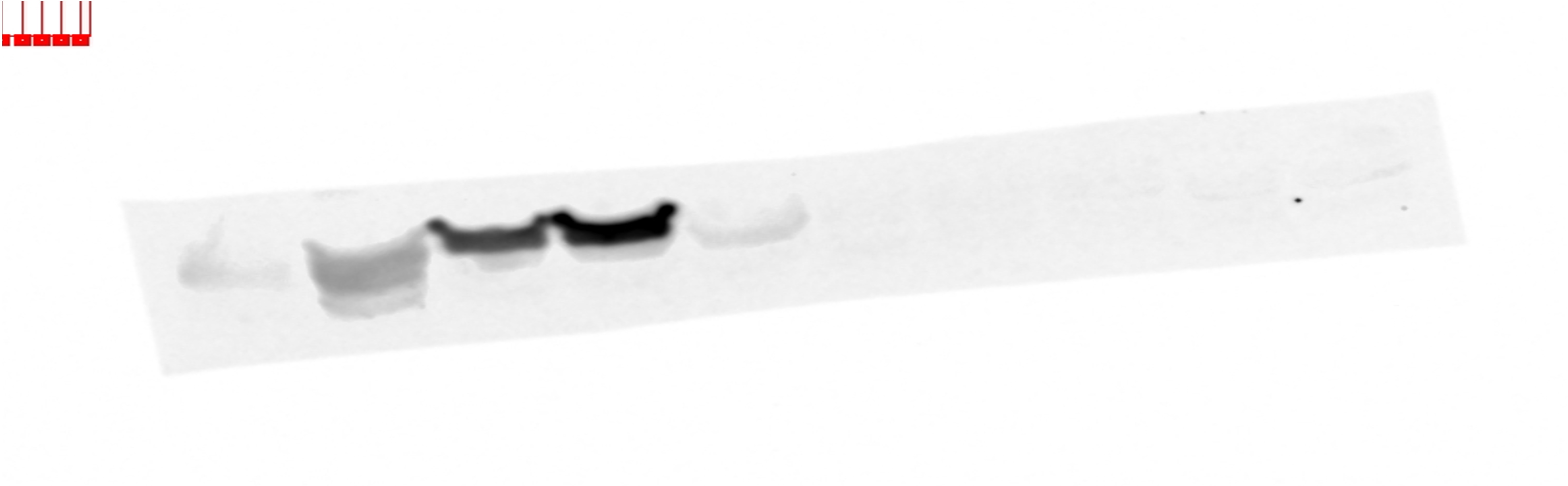

Supplement: Figure 3—figure supplement 1—source data 2. [file elife-104123-fig3-figsupp1-data2.zip › Figure 3-figure supplement 1-Source Data 2/Figure 3-figure supplement 1F-Kbhb western blot.jpg]

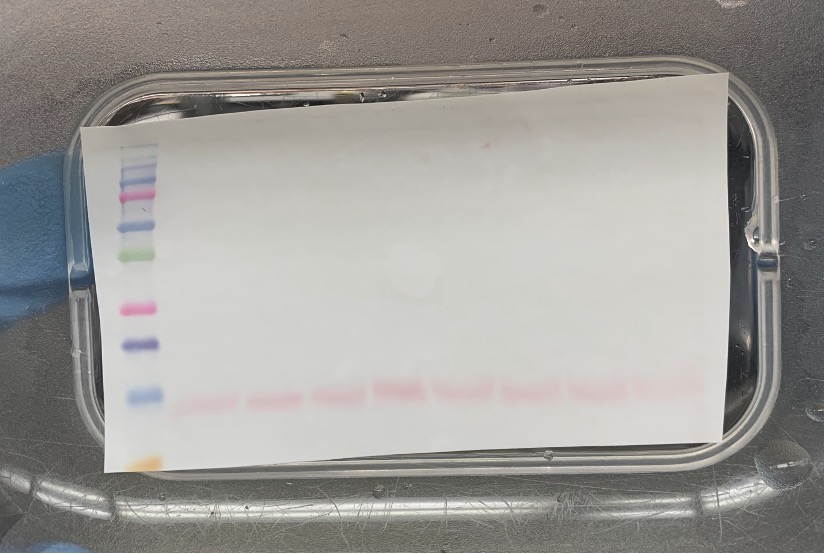

Supplement: Figure 3—figure supplement 1—source data 2. [file elife-104123-fig3-figsupp1-data2.zip › Figure 3-figure supplement 1-Source Data 2/Figure 3-figure supplement 1C-Ponceau S.jpg]

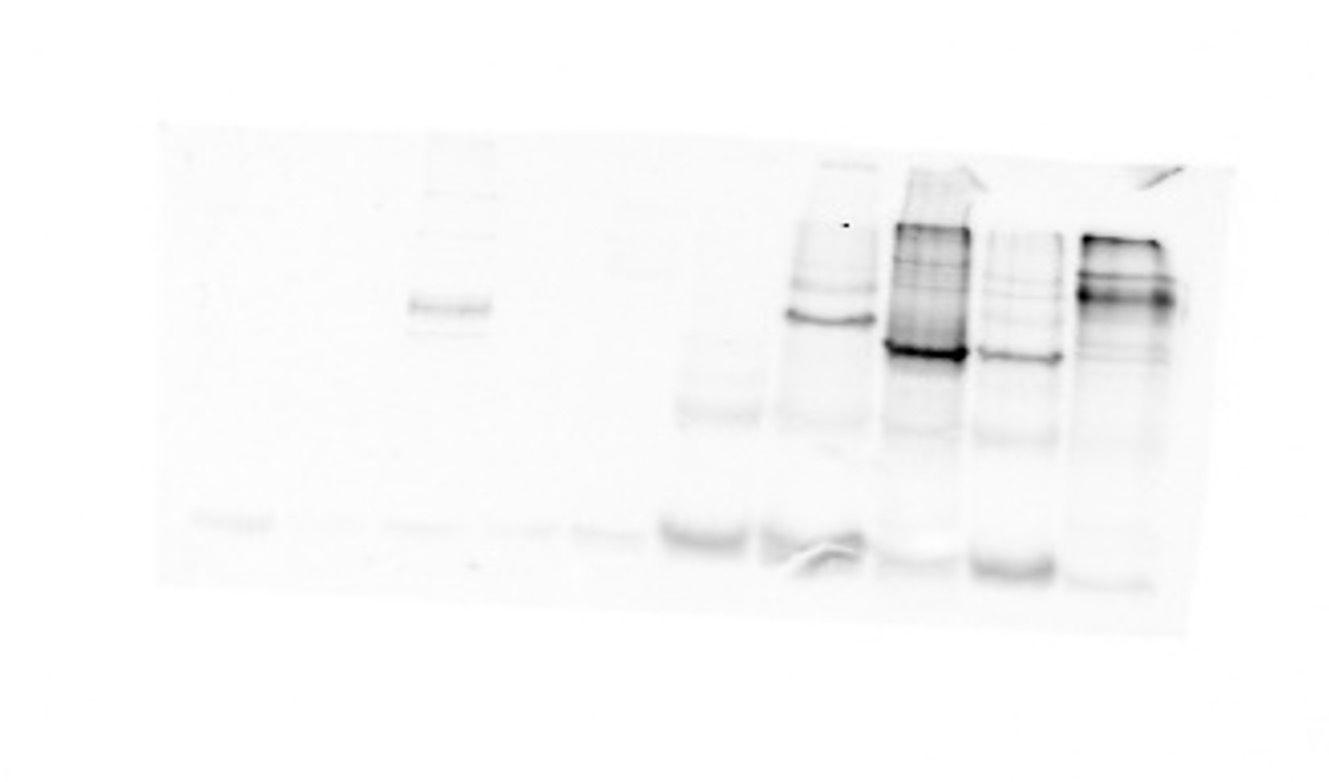

Supplement: Figure 3—figure supplement 1—source data 2. [file elife-104123-fig3-figsupp1-data2.zip › Figure 3-figure supplement 1-Source Data 2/Figure 3-figure supplement 1D-Kbhb western blot.jpg]

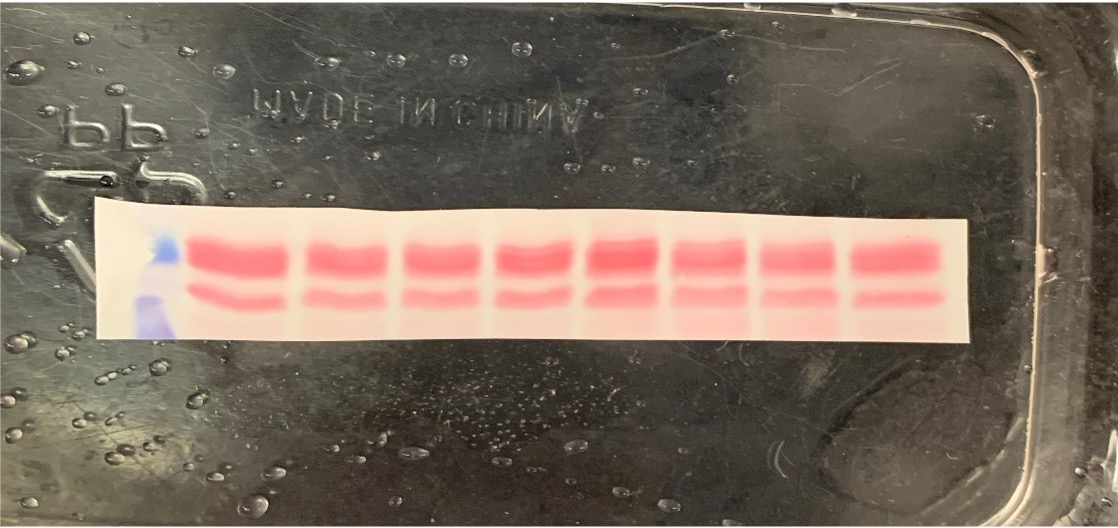

Supplement: Figure 3—figure supplement 1—source data 2. [file elife-104123-fig3-figsupp1-data2.zip › Figure 3-figure supplement 1-Source Data 2/Figure 3-figure supplement 1G-Ponceau S.jpg]

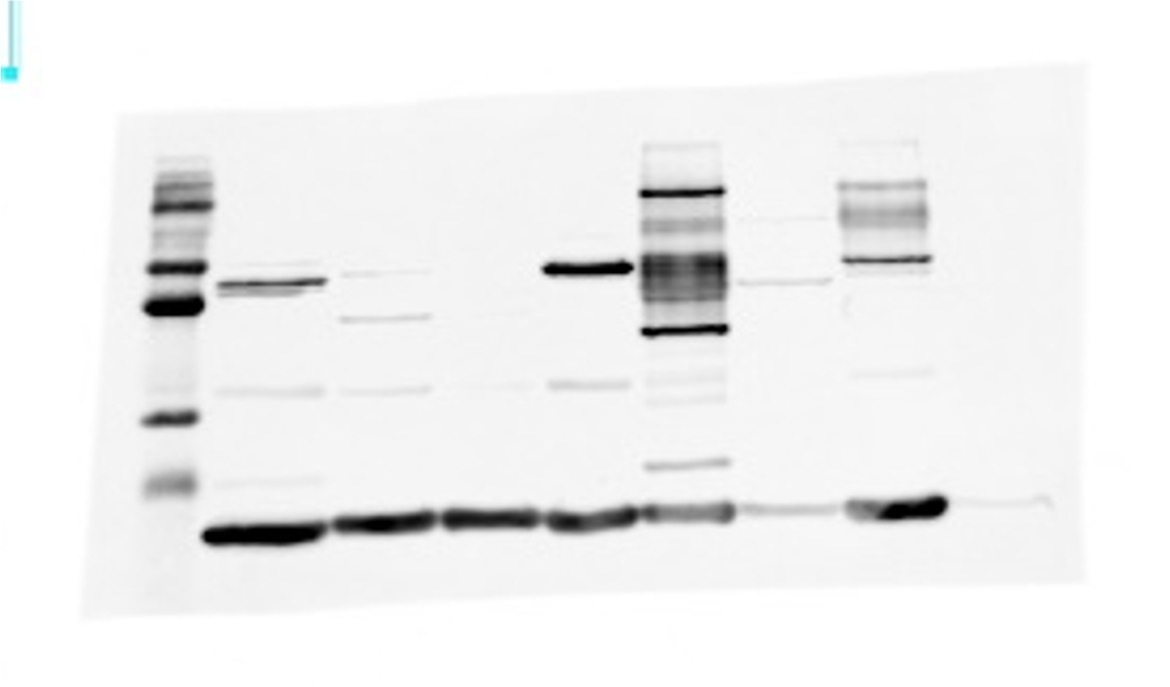

Supplement: Figure 3—figure supplement 1—source data 2. [file elife-104123-fig3-figsupp1-data2.zip › Figure 3-figure supplement 1-Source Data 2/Figure 3-figure supplement 1B-Kac western blot.jpg]

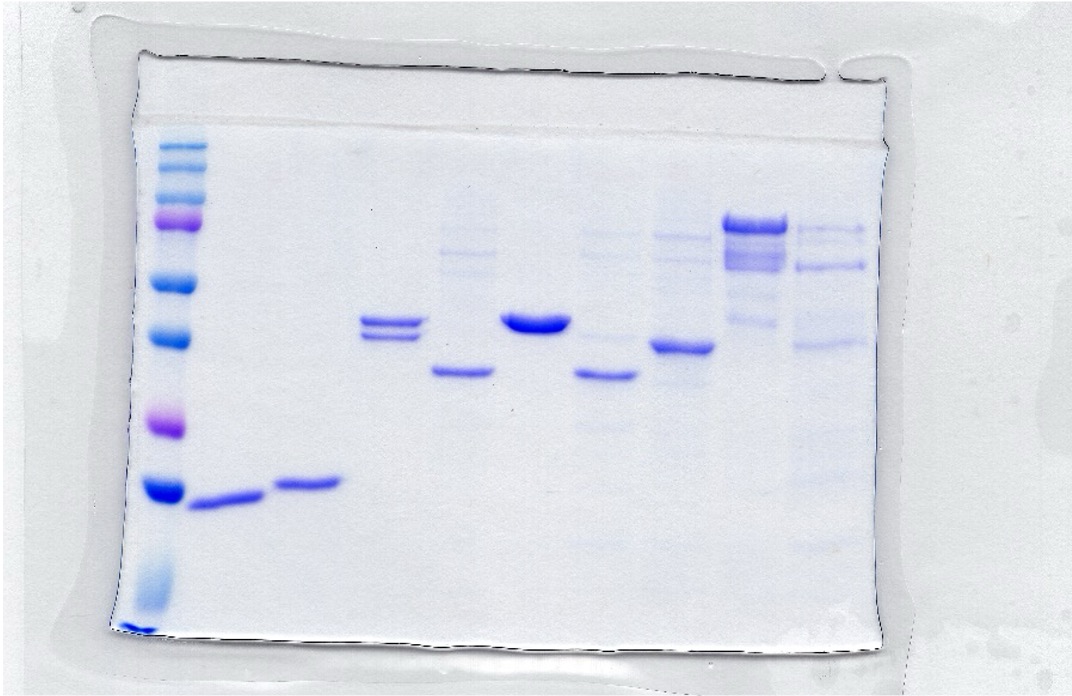

Supplement: Figure 3—figure supplement 1—source data 2. [file elife-104123-fig3-figsupp1-data2.zip › Figure 3-figure supplement 1-Source Data 2/Figure 3-figure supplement 1A-SDS-PAGE gel.jpg]

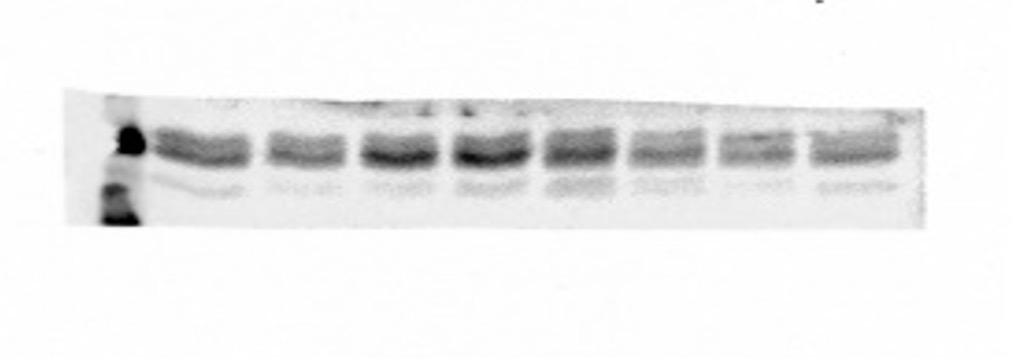

Supplement: Figure 3—figure supplement 1—source data 2. [file elife-104123-fig3-figsupp1-data2.zip › Figure 3-figure supplement 1-Source Data 2/Figure 3-figure supplement 1G-Kbhb western blot.jpg]

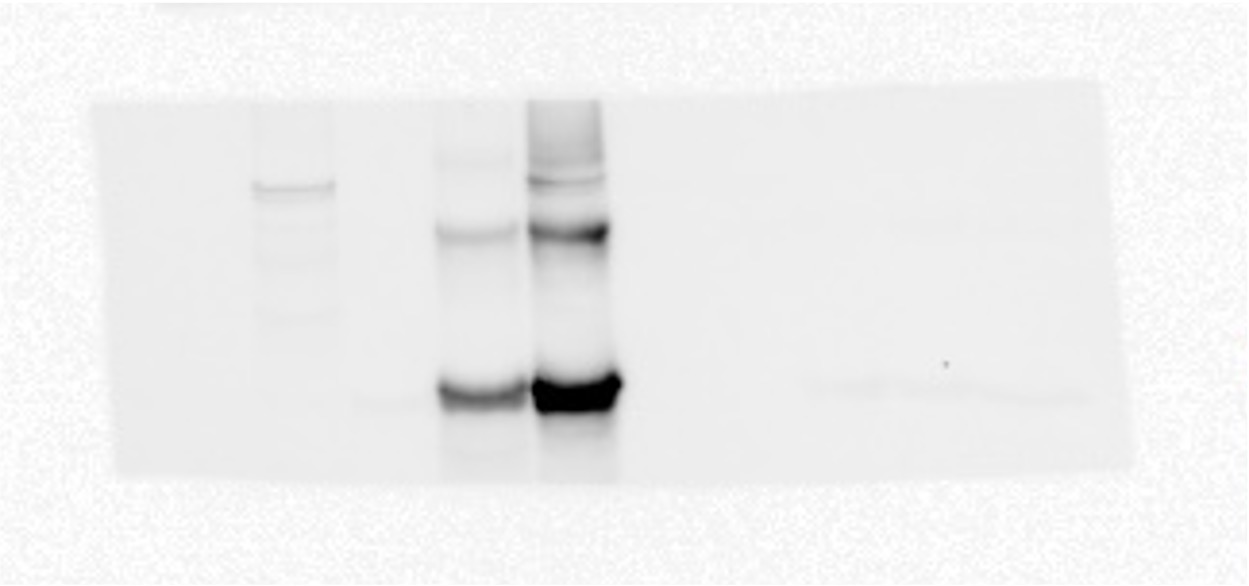

Supplement: Figure 3—figure supplement 1—source data 2. [file elife-104123-fig3-figsupp1-data2.zip › Figure 3-figure supplement 1-Source Data 2/Figure 3-figure supplement 1E-Kbhb western blot.jpg]

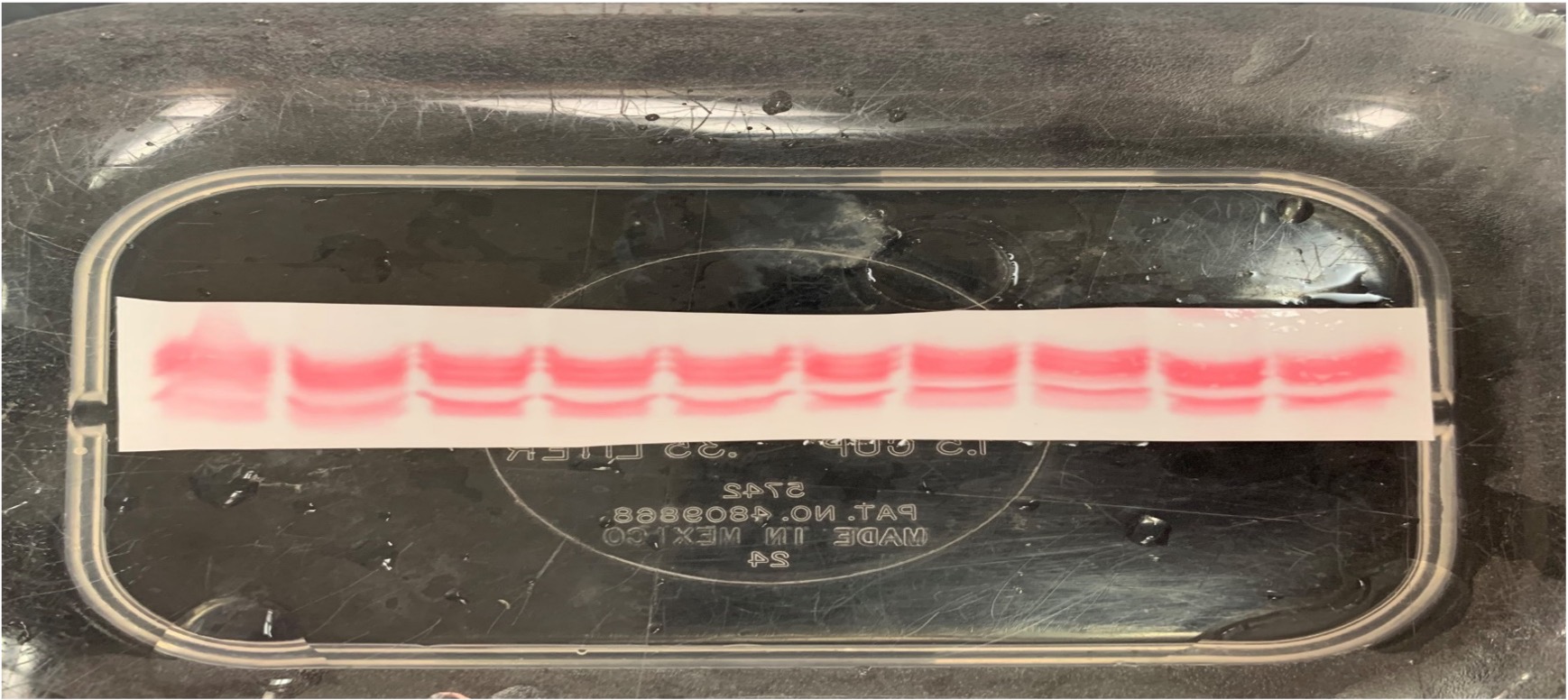

Supplement: Figure 3—figure supplement 1—source data 2. [file elife-104123-fig3-figsupp1-data2.zip › Figure 3-figure supplement 1-Source Data 2/Figure 3-figure supplement 1F-Ponceau S.jpg]

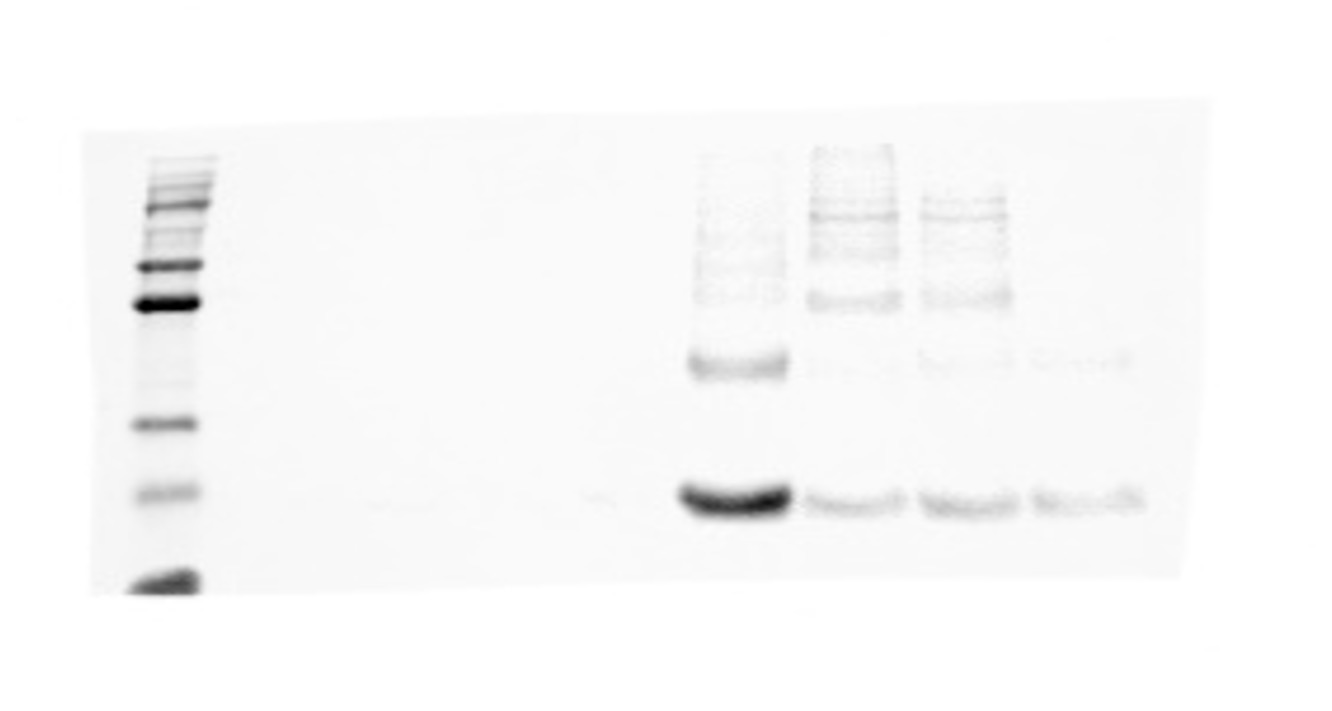

Supplement: Figure 3—figure supplement 1—source data 2. [file elife-104123-fig3-figsupp1-data2.zip › Figure 3-figure supplement 1-Source Data 2/Figure 3-figure supplement 1C-Kbhb western blot.jpg]

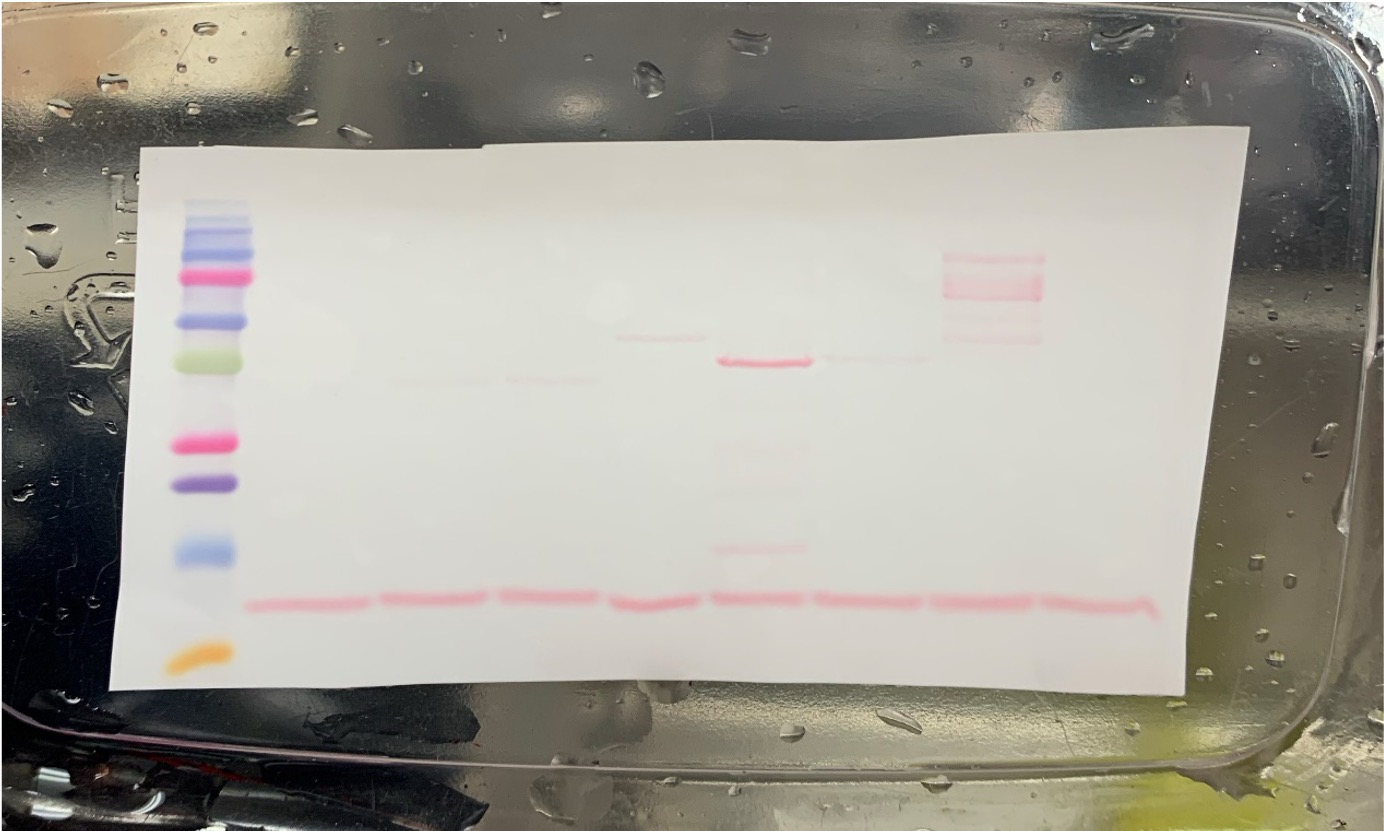

Supplement: Figure 3—figure supplement 1—source data 2. [file elife-104123-fig3-figsupp1-data2.zip › Figure 3-figure supplement 1-Source Data 2/Figure 3-figure supplement 1B-Ponceau S.jpg]

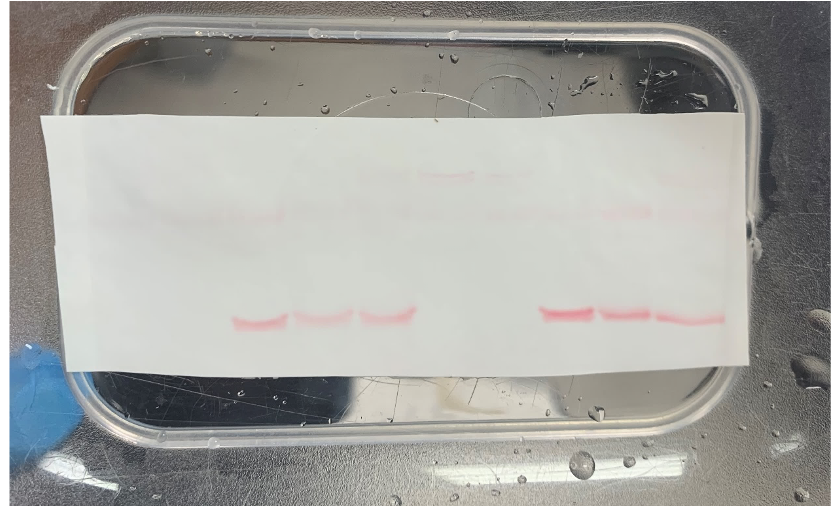

Supplement: Figure 3—figure supplement 1—source data 2. [file elife-104123-fig3-figsupp1-data2.zip › Figure 3-figure supplement 1-Source Data 2/Figure 3-figure supplement 1E-Ponceau S.jpg]

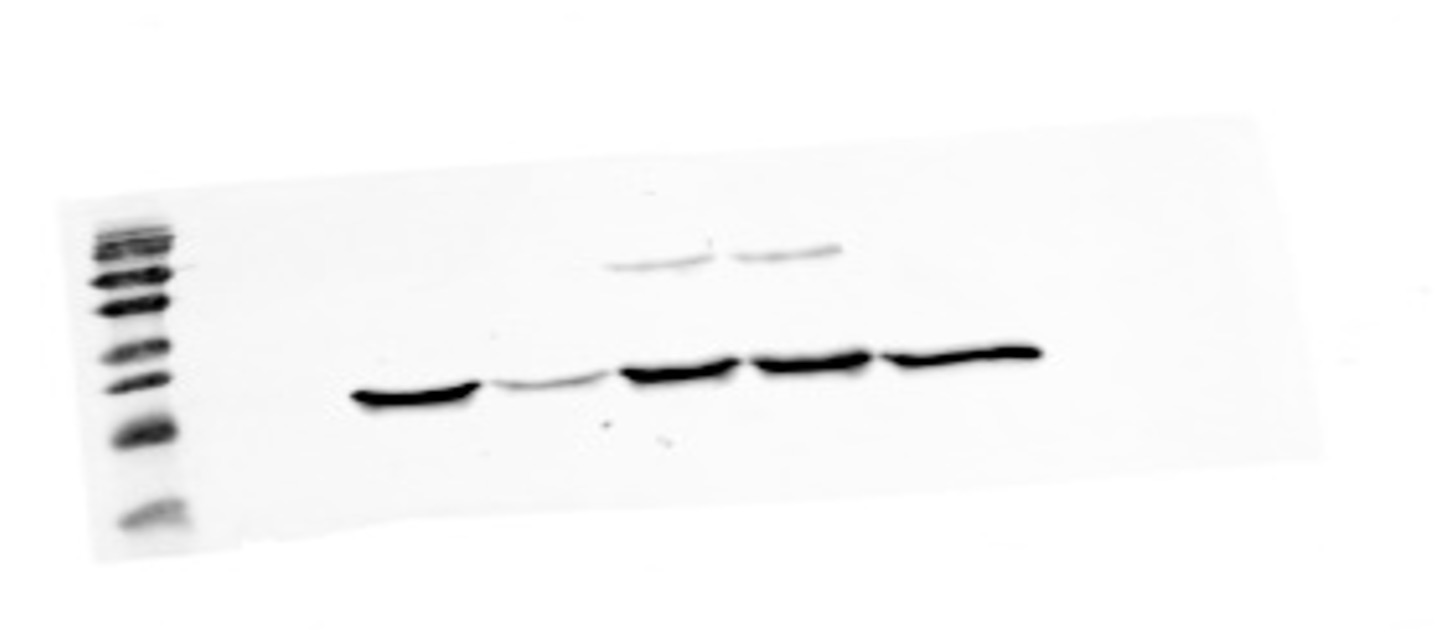

Supplement: Figure 3—figure supplement 2—source data 2. [file elife-104123-fig3-figsupp2-data2.zip › Figure 3-figure supplement 2-Source Data 2/Figure 3-figure supplement 2D-NaBH4+ western blot.jpg]

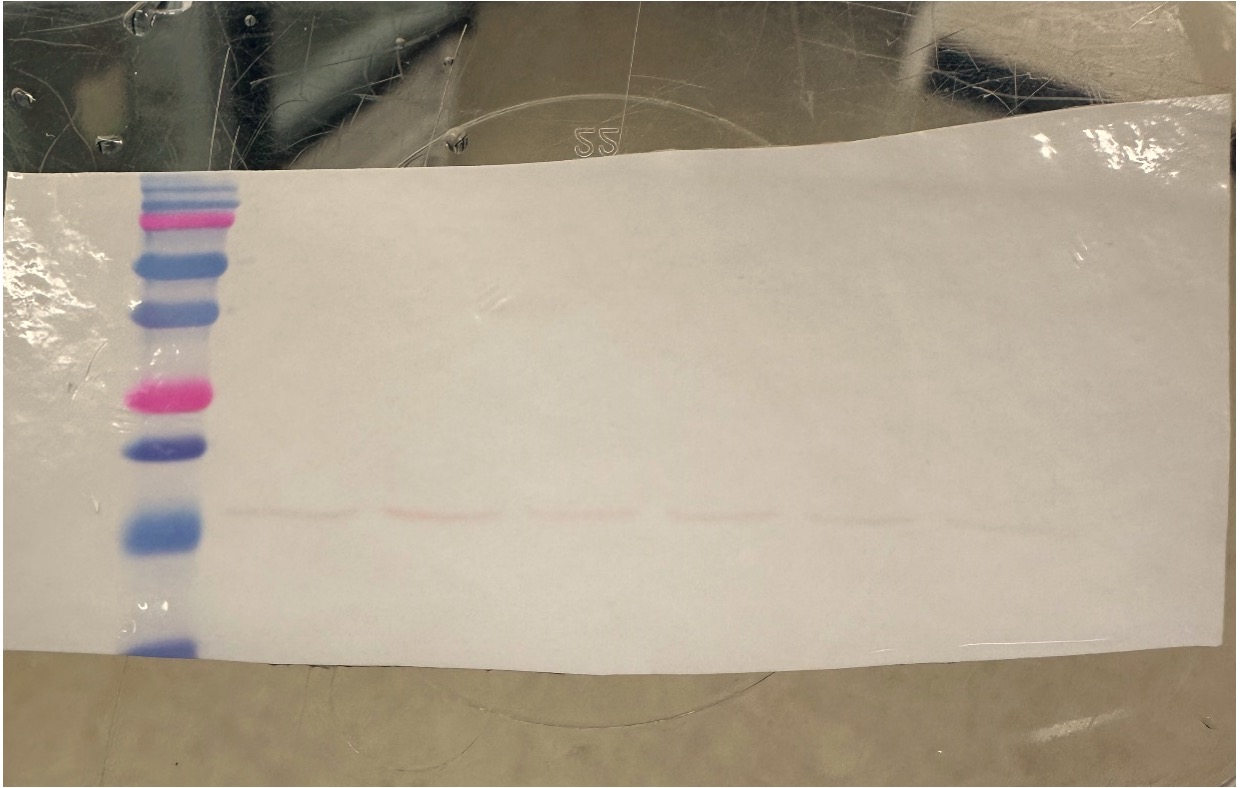

Supplement: Figure 3—figure supplement 2—source data 2. [file elife-104123-fig3-figsupp2-data2.zip › Figure 3-figure supplement 2-Source Data 2/Figure 3-figure supplement 2D-Ponceau S.jpg]

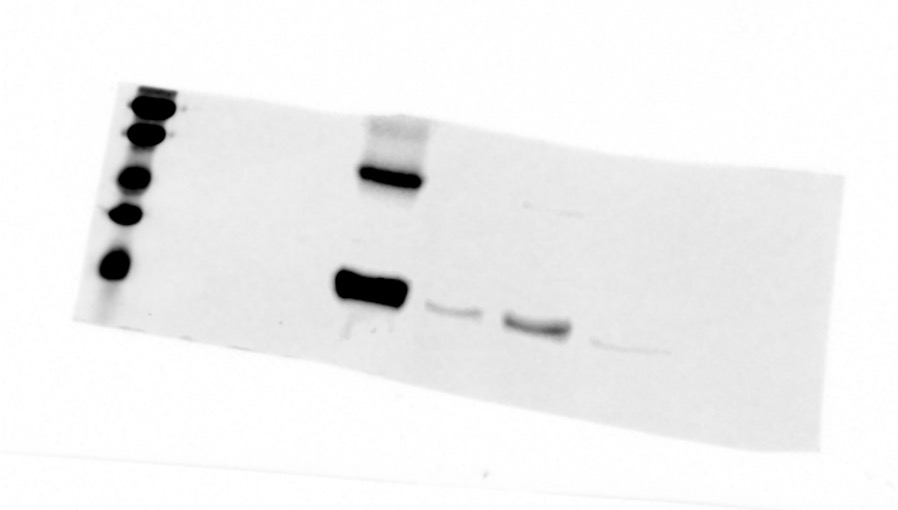

Supplement: Figure 3—figure supplement 2—source data 2. [file elife-104123-fig3-figsupp2-data2.zip › Figure 3-figure supplement 2-Source Data 2/Figure 3-figure supplement 2B-Kbhb western blot.jpg]

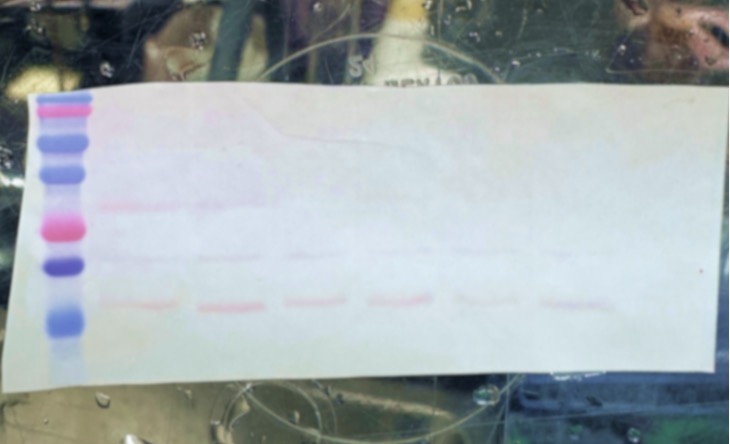

Supplement: Figure 3—figure supplement 2—source data 2. [file elife-104123-fig3-figsupp2-data2.zip › Figure 3-figure supplement 2-Source Data 2/Figure 3-figure supplement 2B-Ponceau S.jpg]

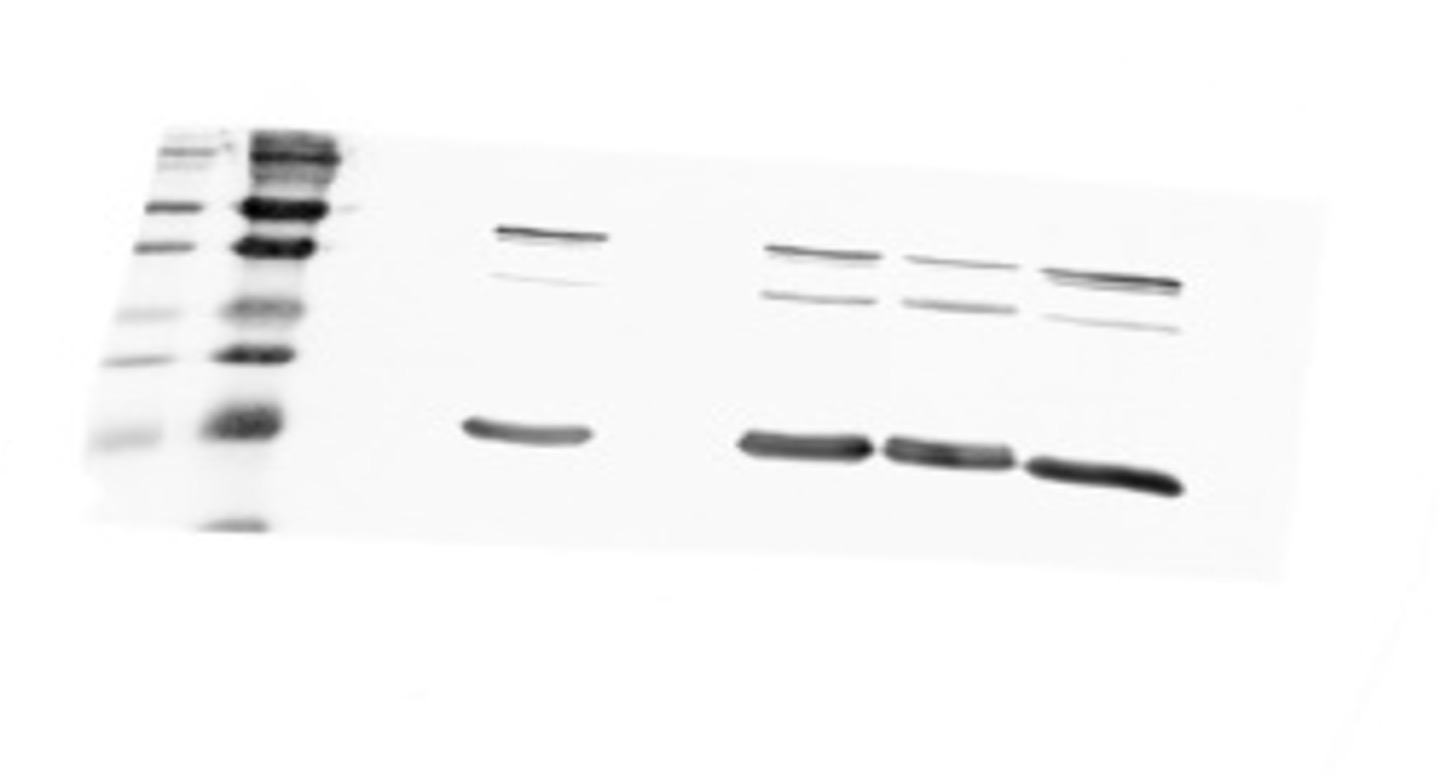

Supplement: Figure 3—figure supplement 2—source data 2. [file elife-104123-fig3-figsupp2-data2.zip › Figure 3-figure supplement 2-Source Data 2/Figure 3-figure supplement 2C-Kac western blot.jpg]

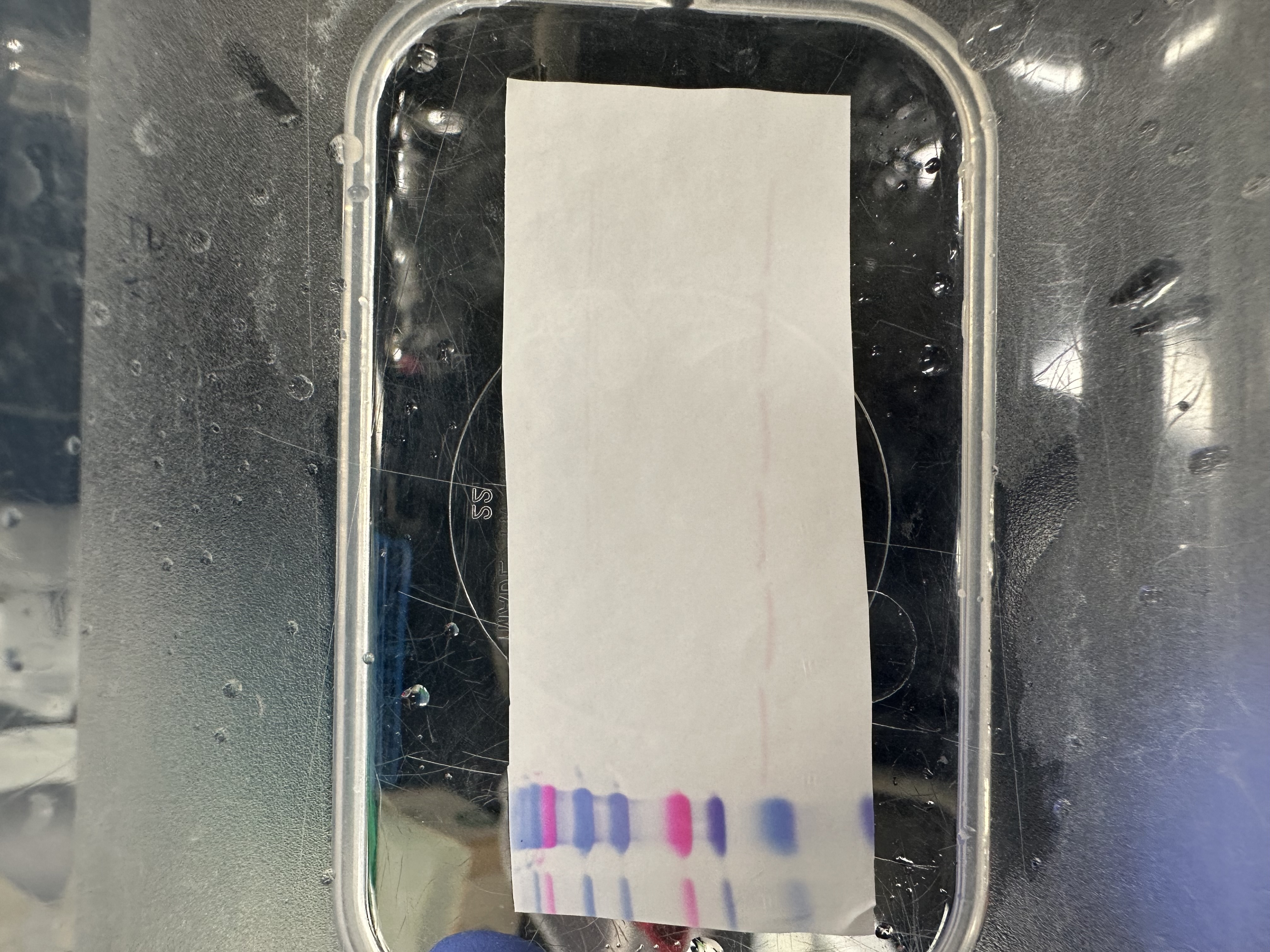

Supplement: Figure 3—figure supplement 2—source data 2. [file elife-104123-fig3-figsupp2-data2.zip › Figure 3-figure supplement 2-Source Data 2/Figure 3-figure supplement 2C-Ponceau S.jpeg]

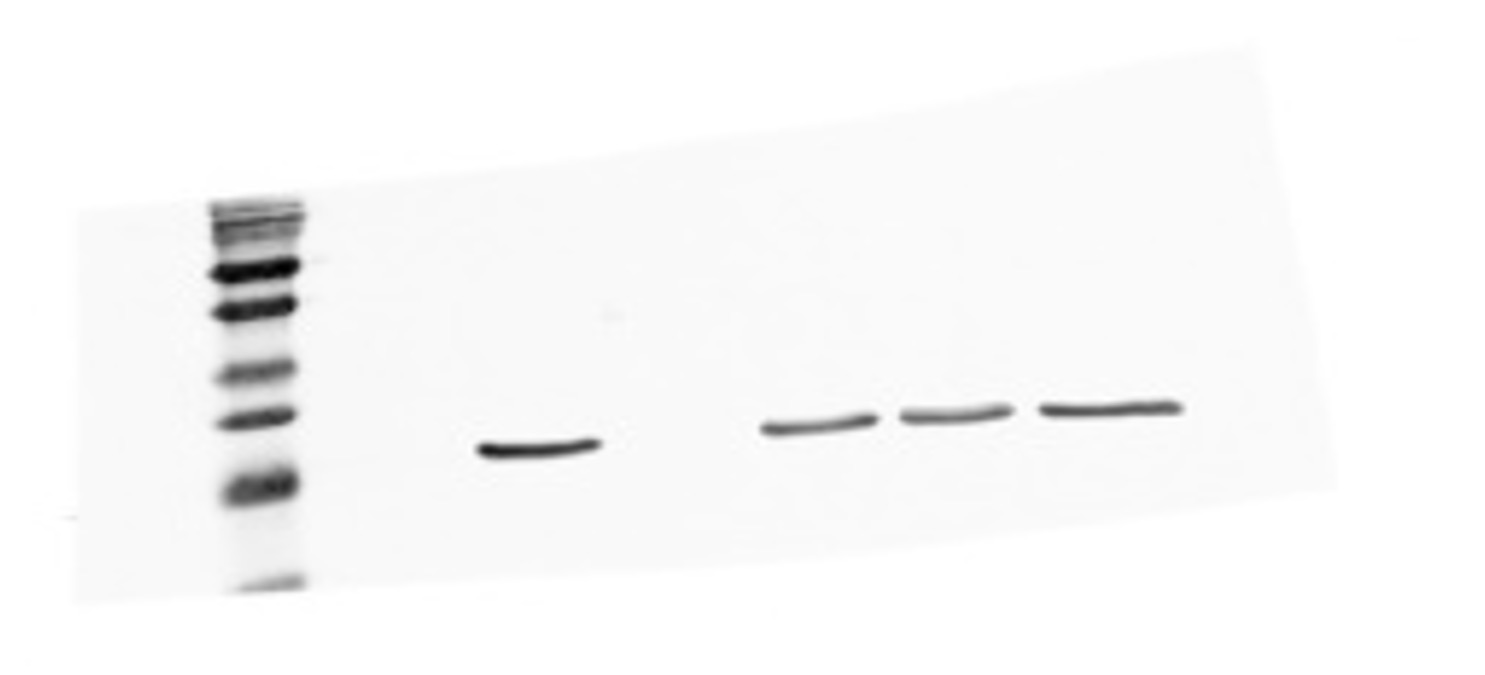

Supplement: Figure 3—figure supplement 2—source data 2. [file elife-104123-fig3-figsupp2-data2.zip › Figure 3-figure supplement 2-Source Data 2/Figure 3-figure supplement 2D-NaBH4- western blot.jpg]

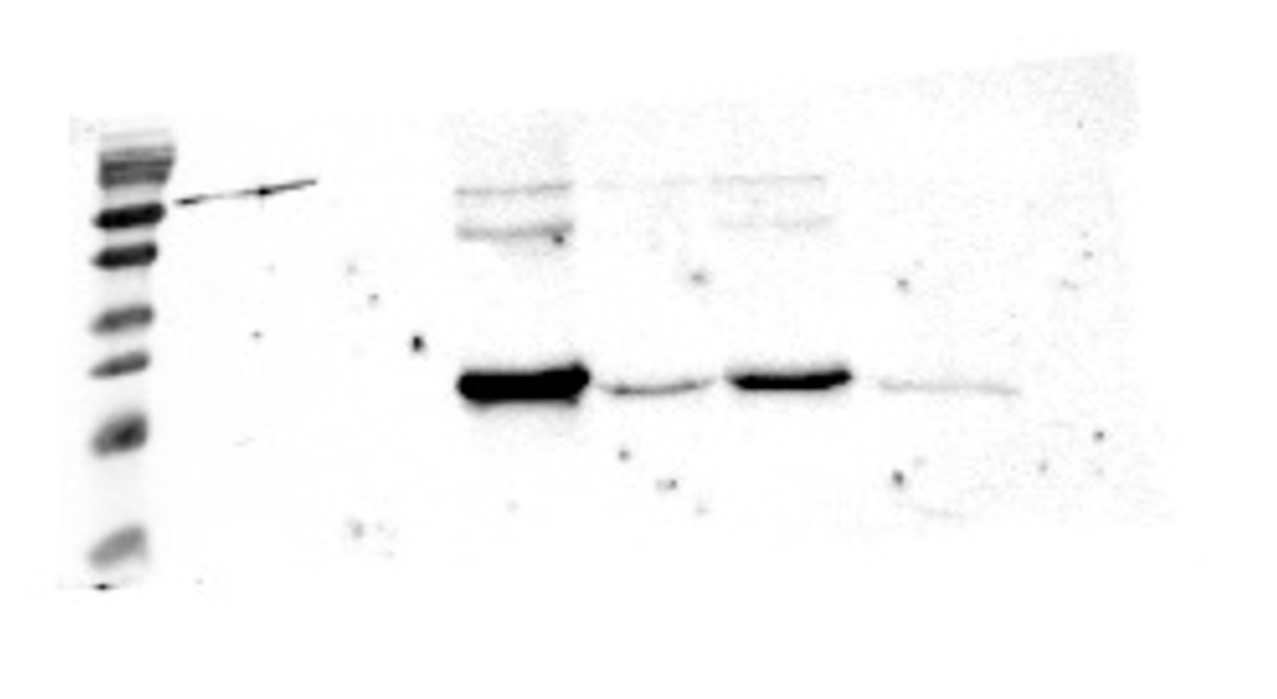

Supplement: Figure 3—figure supplement 2—source data 2. [file elife-104123-fig3-figsupp2-data2.zip › Figure 3-figure supplement 2-Source Data 2/Figure 3-figure supplement 2C-Kbhb western blot.jpg]

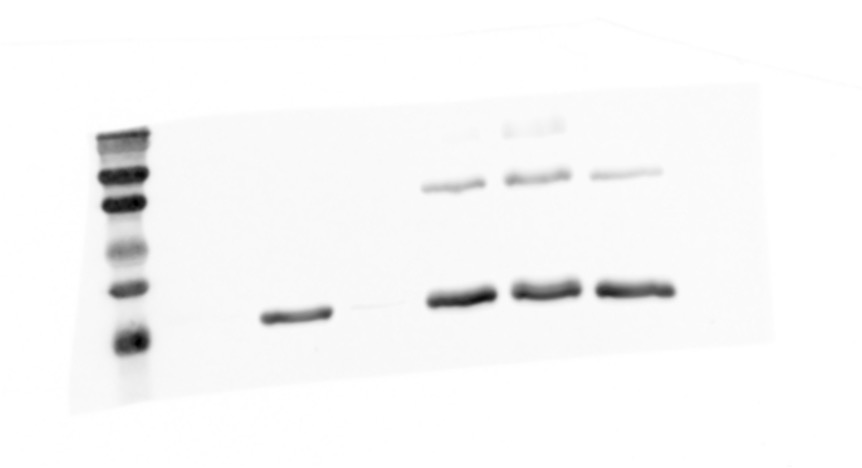

Supplement: Figure 3—figure supplement 2—source data 2. [file elife-104123-fig3-figsupp2-data2.zip › Figure 3-figure supplement 2-Source Data 2/Figure 3-figure supplement 2B-Kac western blot.jpg]
