# Supplementary material for: Identification of the regulatory elements and protein substrates of lysine acetoacetylation
Source: eLife. 2026 May 14;14:RP104123. doi: 10.7554/eLife.104123 (PMC13175576; doi:10.7554/eLife.104123)
Supplement: Figure 2—source data 1. [file elife-104123-fig2-data1.pdf]

Detected by blotting with anti-Kbhb

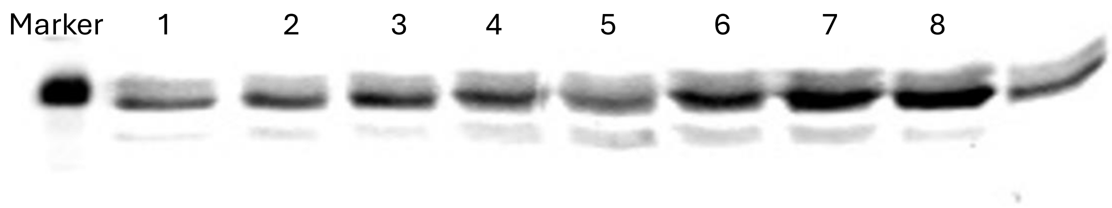

Ponceau staining

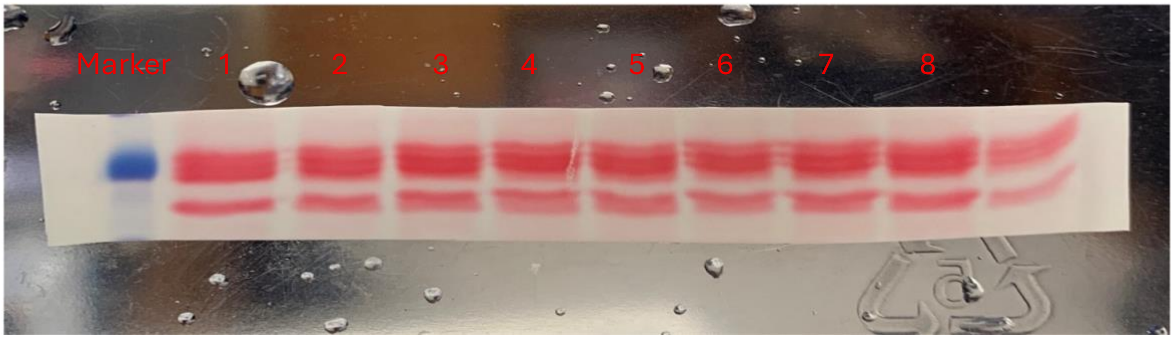

Detected by blotting with anti-H3

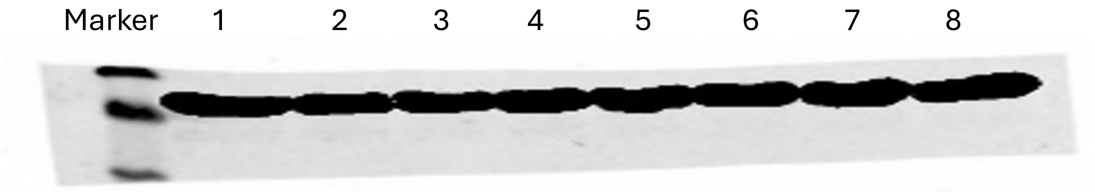

Figure 2, Source Data 1. (A) Original membranes corresponding to Figure 2, panel A. Lanes 1–4 show non–$NaBH_4$-reduced histone samples from HEK293T cells treated with 0, 5, 10, or 20 mM acetoacetate, whereas lanes 5–8 show the corresponding $NaBH_4$-reduced histone samples treated under the same conditions.

Detected by blotting with anti-Kbhb

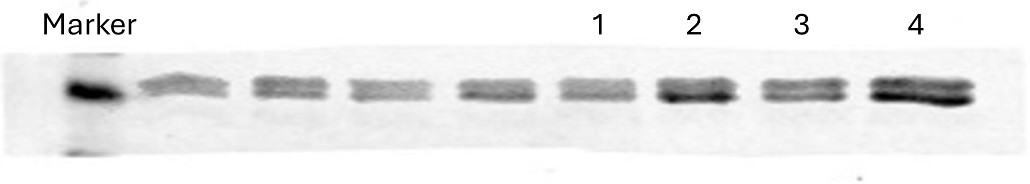

Ponceau staining

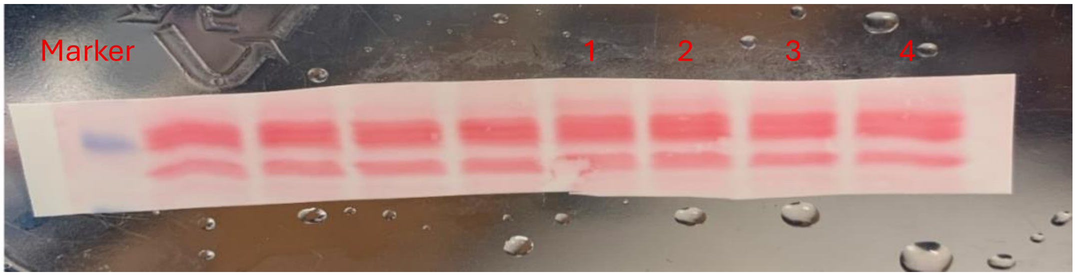

Figure 2, Source Data 1. (B) Original membranes corresponding to Figure 2, panel B. Lanes 1–2 show non–$NaBH_4$-reduced histone samples from HCT116 cells treated with 0 or 20 mM acetoacetate, whereas lanes 3–4 show the corresponding $NaBH_4$-reduced histone samples under the same conditions.

Detected by blotting with anti-Kbhb

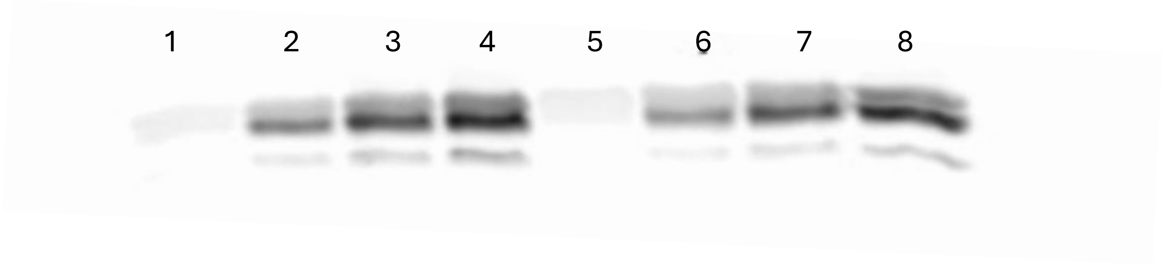

Ponceau staining

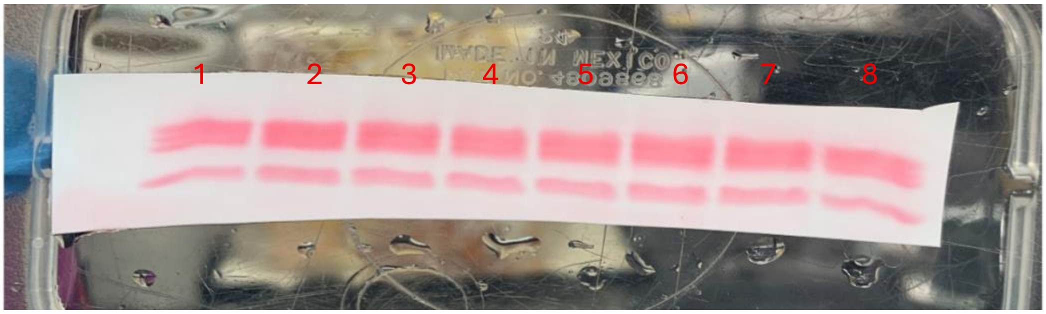

Figure 2, Source Data 1. (C) Original membranes corresponding to Figure 2, panel C. Lanes 1–4 show non–$NaBH_4$-reduced histone samples from HEK293T cells treated with 0, 5, 10, or 20 mM β-hydroxybutyrate, while lanes 5–8 show $NaBH_4$-reduced histone samples from HEK293T cells treated with the same concentrations.

Detected by blotting with anti-Kbhb

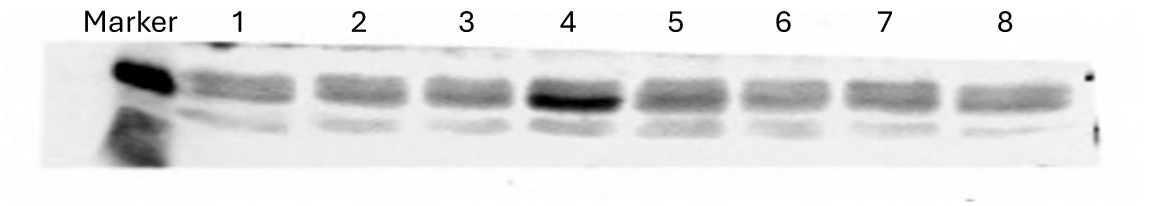

Ponceau staining

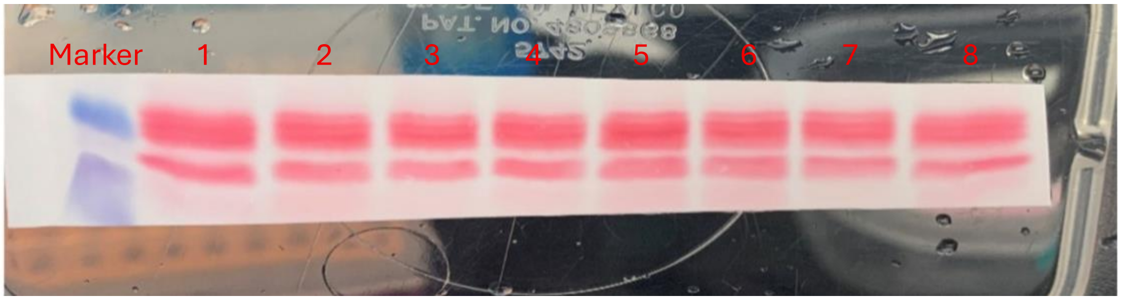

Figure 2, Source Data 1. (D) Original membranes corresponding to Figure 2, panel D. Lanes 1–4 show $NaBH_4$-reduced histone samples from HEK293T cells with no treatment or treated with leucine, lysine, or acetoacetate, whereas lanes 5–8 show the corresponding non–$NaBH_4$-reduced histone samples under the same conditions.

Detected by blotting with anti-Kbhb

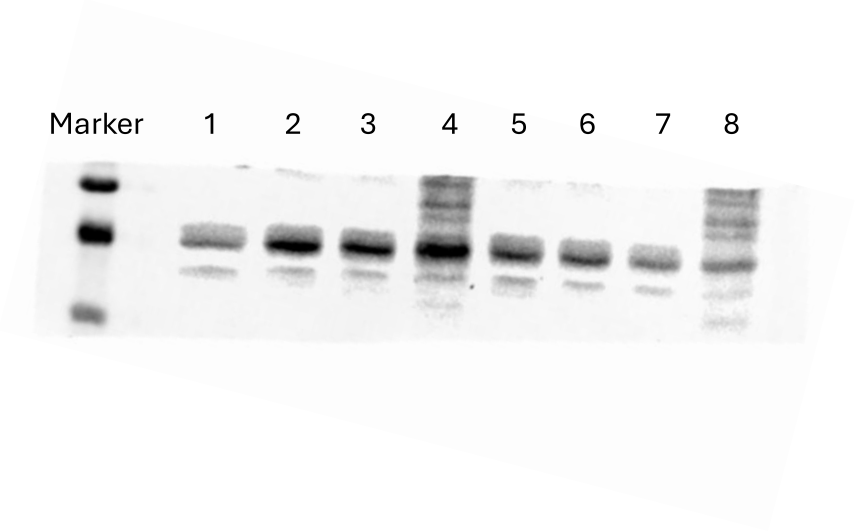

Ponceau staining

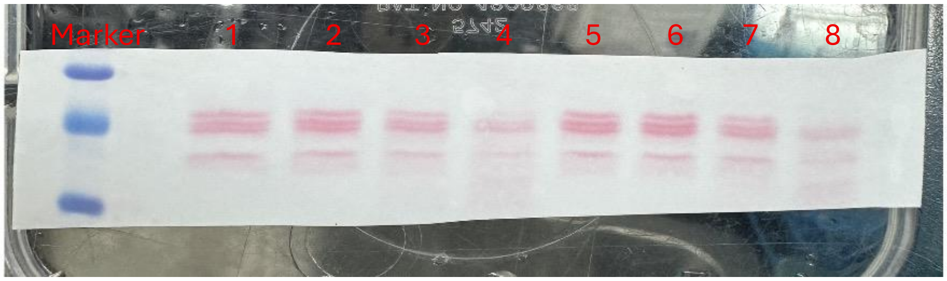

Detected by blotting with anti-H3

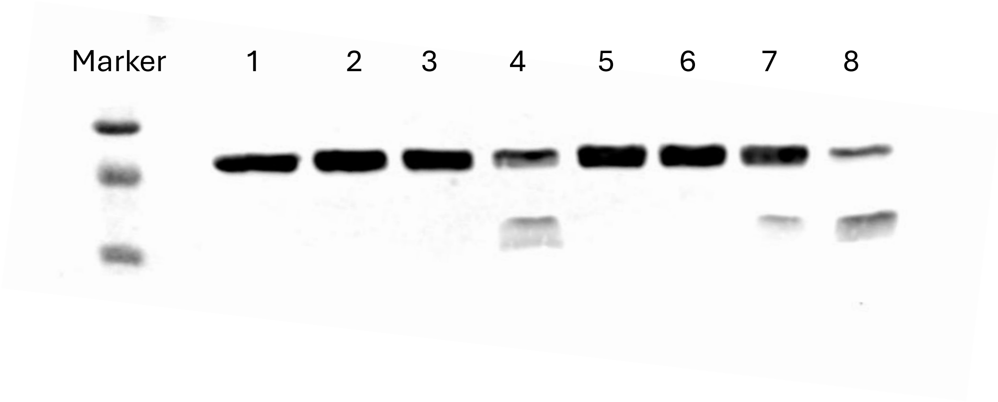

Figure 2, Source Data 1. (E) Original membranes corresponding to Figure 2, panel E. Lanes 1–4 show $NaBH_4$-reduced histone samples from HEK293T cells subjected to no treatment, acetoacetate treatment, SCOT overexpression plus acetoacetate treatment, or AACS overexpression plus acetoacetate treatment, while lanes 5–8 show the corresponding non–$NaBH_4$-reduced histone samples under the same conditions.

Detected by blotting with anti-Kbhb

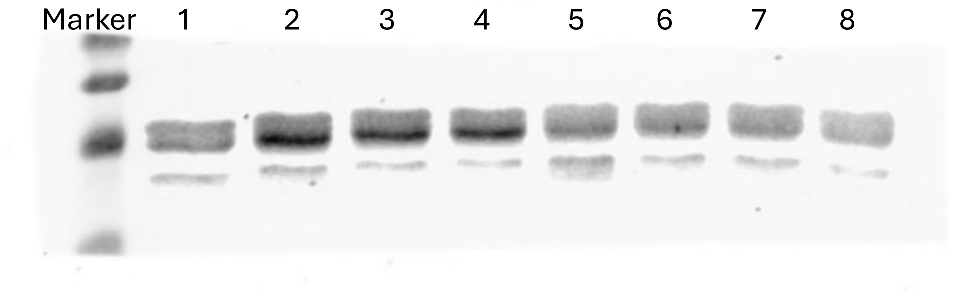

Ponceau staining

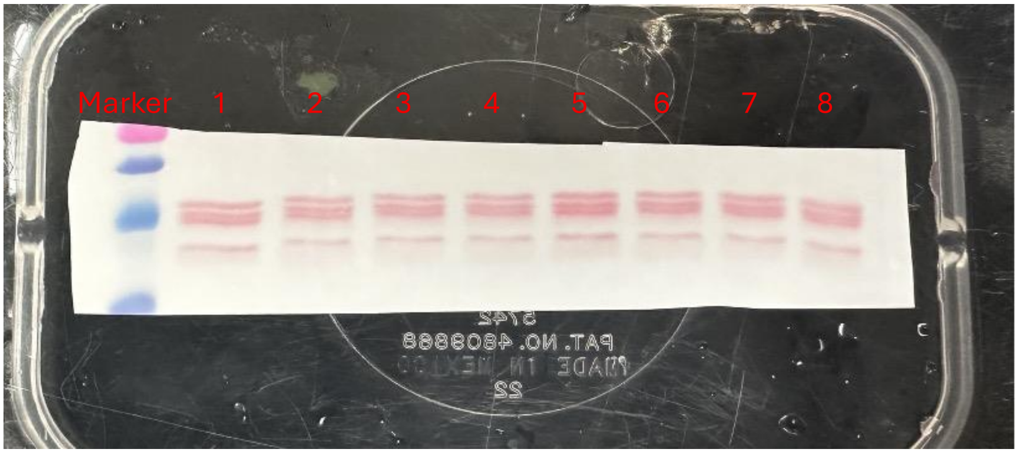

Detected by blotting with anti-H3

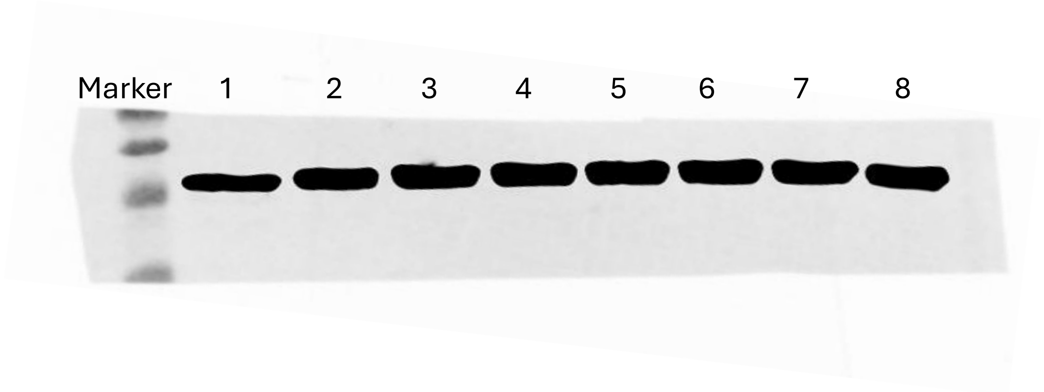

Figure 2, Source Data 1. (F) Original membranes corresponding to Figure 2, panel F. Lanes 1–4 show $NaBH_4$-reduced histone samples from HEK293T cells subjected to no treatment, acetoacetate, 0.1 mM AHA plus acetoacetate, or 0.5 mM AHA plus acetoacetate, whereas lanes 5–8 show the corresponding non–$NaBH_4$-reduced histone samples under the same conditions.
