# Supplementary material for: Identification of the regulatory elements and protein substrates of lysine acetoacetylation
Source: eLife. 2026 May 14;14:RP104123. doi: 10.7554/eLife.104123 (PMC13175576; doi:10.7554/eLife.104123)
Supplement: Figure 2—figure supplement 1—source data 1. [file elife-104123-fig2-figsupp1-data1.pdf]

Detected by blotting with anti-Kbhb

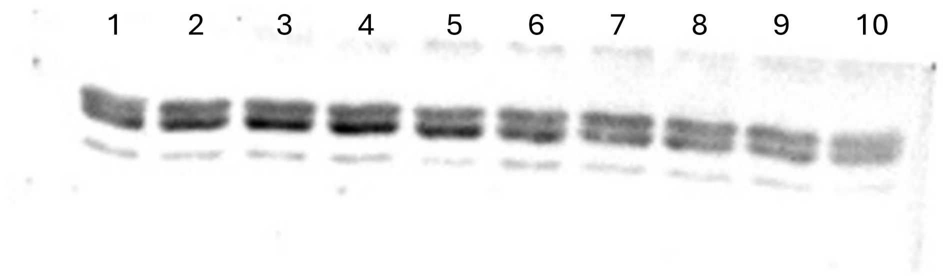

Ponceau staining

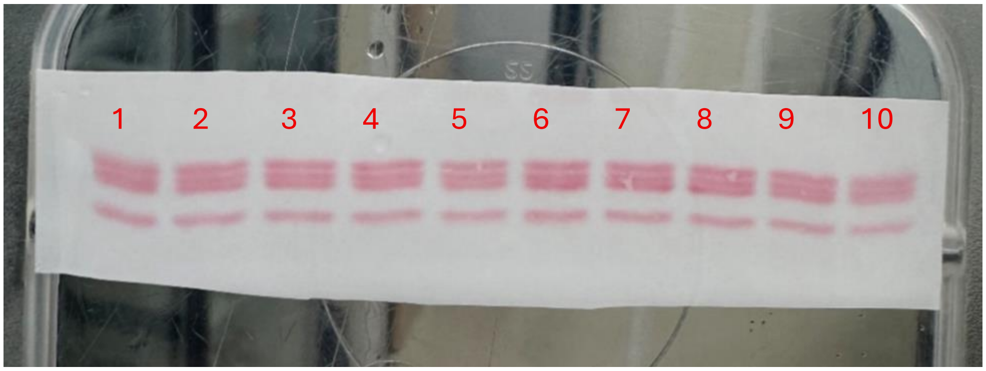

Detected by blotting with anti-H3

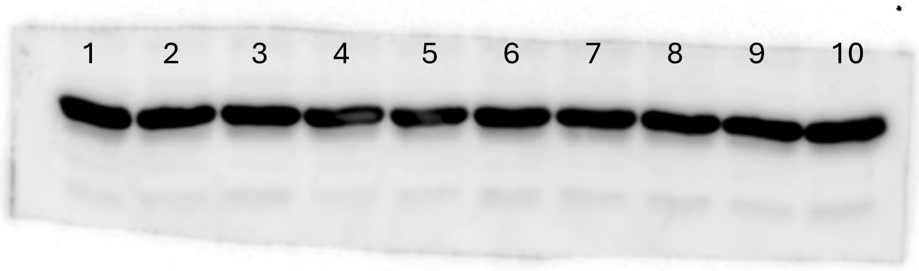

Figure 2-figure supplement 1, Source Data 1. (A) Original membranes corresponding to Figure 2-figure supplement 1, panel A. Lanes 1–5 show $NaBH_4$-reduced histone samples from HEK293T cells treated with acetoacetate for 0, 1, 3, 6, or 24 h, while lanes 6–10 show non-$NaBH_4$-reduced histone samples from HEK293T cells treated with acetoacetate for the same incubation times.

Detected by blotting with anti-flag (left), and anti-actin (right)

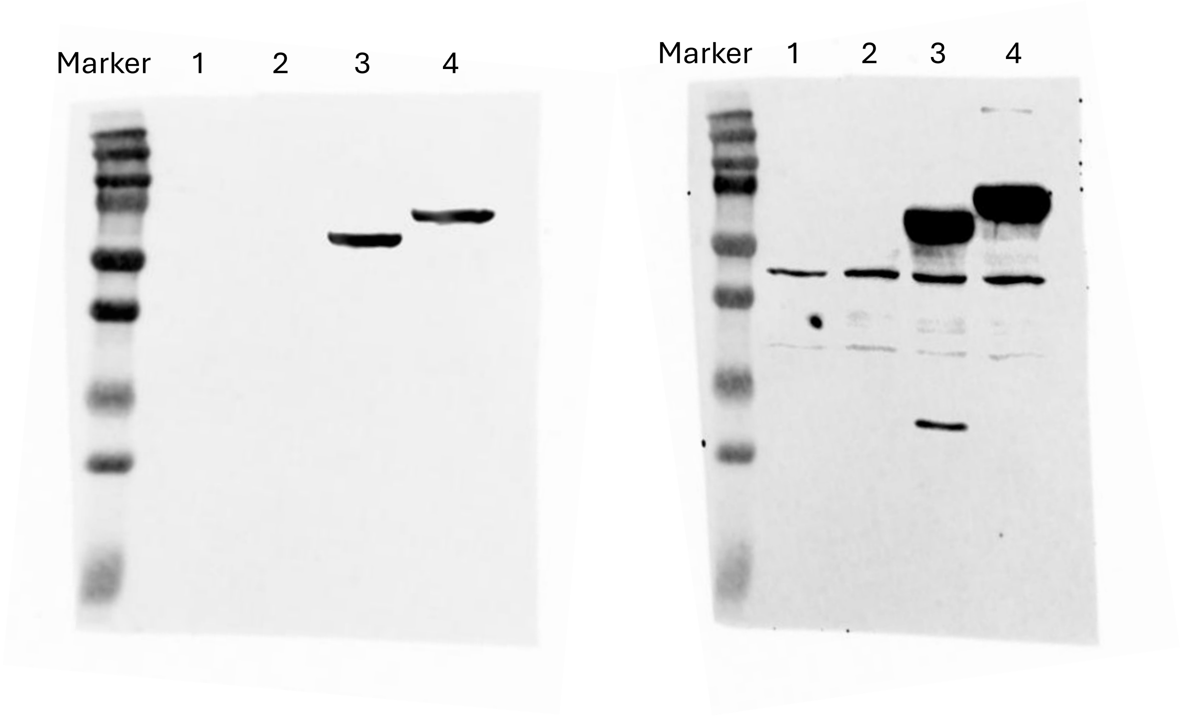

Figure 2-figure supplement 1, Source Data 1. (B) Original membranes corresponding to Figure 2-figure supplement 1, panel B. Lanes 1–4 represent whole proteome samples from HEK293T cells subjected to no treatment, acetoacetate treatment, SCOT overexpression, or AACS overexpression.

Detected by blotting with anti-flag (left), and anti-actin (right)

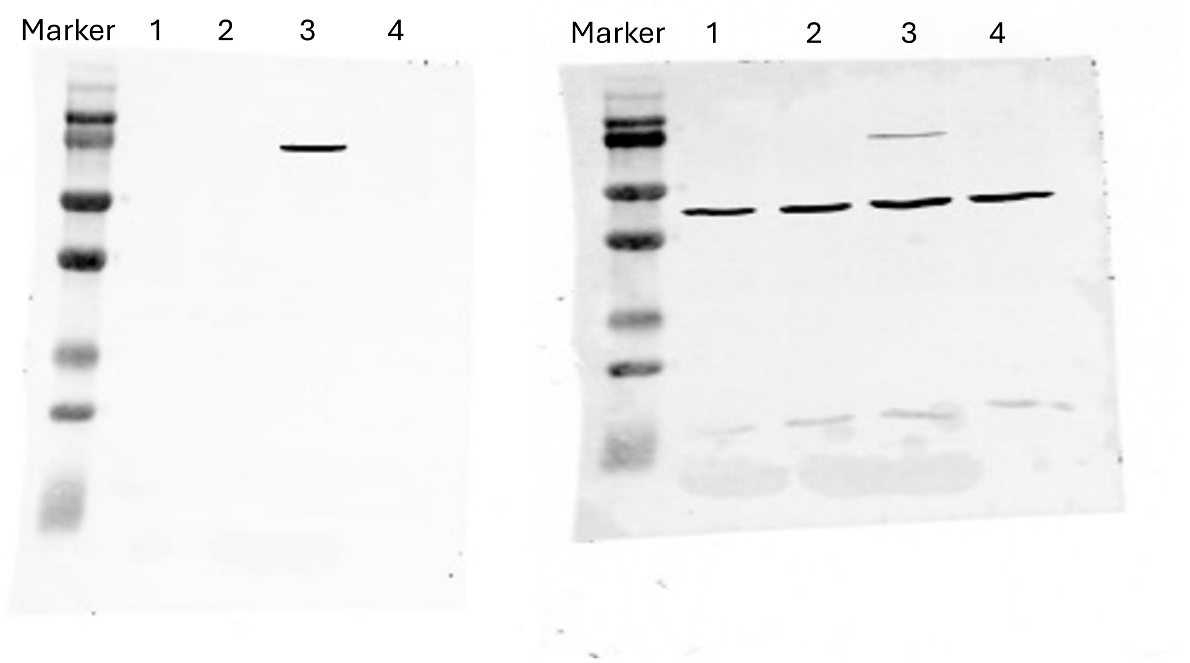

Figure 2-figure supplement 1, Source Data 1. (C) Original membranes corresponding to Figure 2-figure supplement 1, panel C. Lanes 1–4 represent whole proteome samples from HepG2 cells subjected to no treatment, acetoacetate treatment, AACS overexpression plus acetoacetate treatment, or HMGCR overexpression plus acetoacetate treatment.

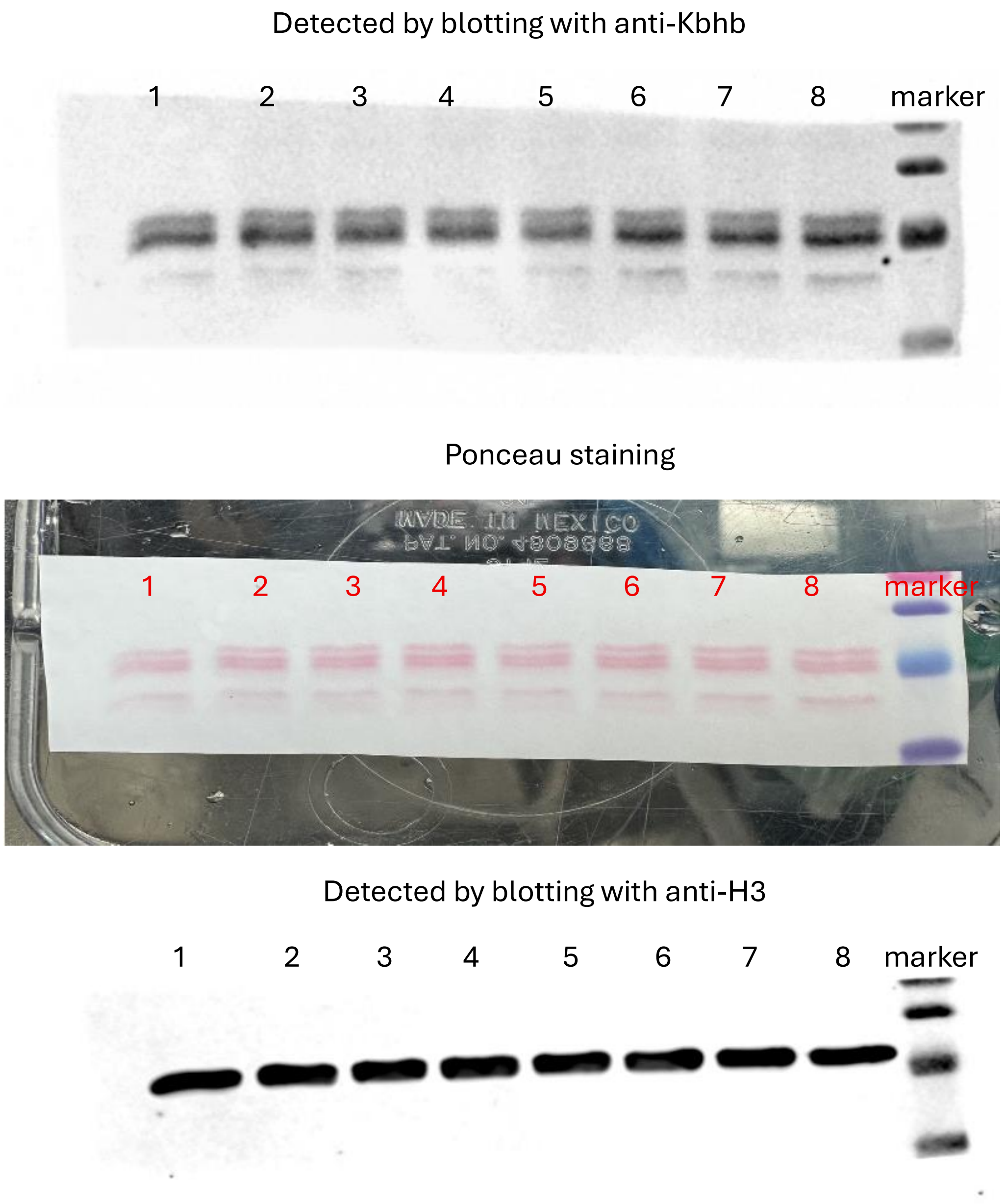

Figure 2-figure supplement 1, Source Data 1. (D) Original membranes corresponding to Figure 2-figure supplement 1, panel D. Lanes 1–4 show non-$NaBH_4$-reduced histone samples from HepG2 cells subjected to no treatment, acetoacetate treatment, AACS overexpression plus acetoacetate treatment, or HMGCR overexpression plus acetoacetate treatment, while lanes 6–10 show $NaBH_4$-reduced histone samples from HepG2 cells with same treatments.

Detected by blotting with anti-Kbhb

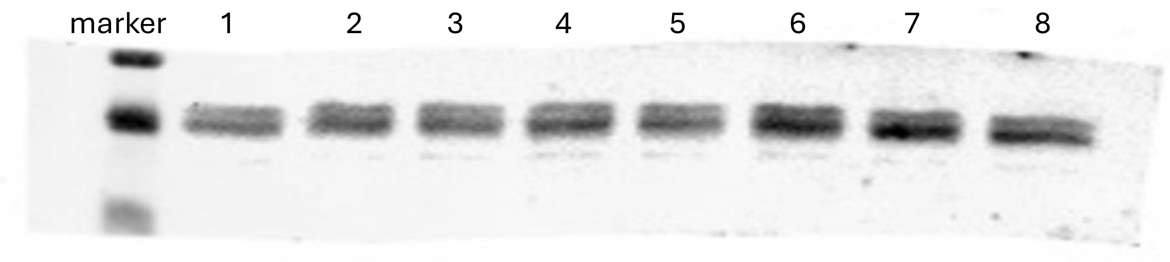

Ponceau staining

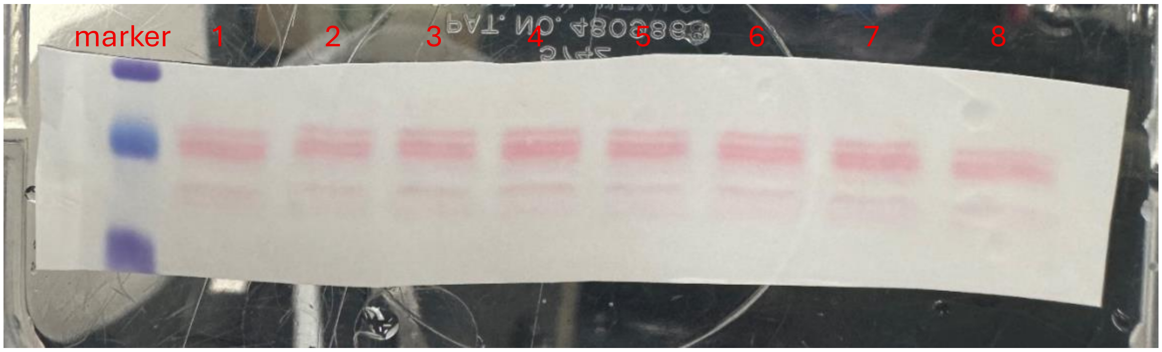

Detected by blotting with anti-H3

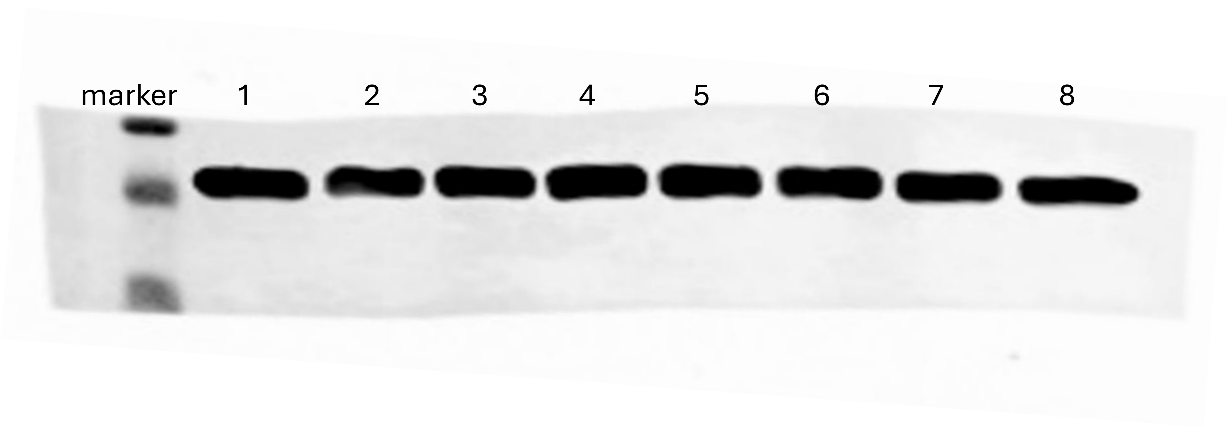

Figure 2-figure supplement 1, Source Data 1. (E) Original membranes corresponding to Figure 2-figure supplement 1, panel E. Lanes 1–4 show non-$NaBH_4$-reduced histone samples from HepG2 cells subjected to no treatment, acetoacetate treatment, 4 uM lovastatin plus acetoacetate treatment, or 20 uM lovastatin plus acetoacetate treatment, while lanes 6–10 show $NaBH_4$-reduced histone samples from HepG2 cells with same treatments.
