# Supplementary material for: Identification of the regulatory elements and protein substrates of lysine acetoacetylation
Source: eLife. 2026 May 14;14:RP104123. doi: 10.7554/eLife.104123 (PMC13175576; doi:10.7554/eLife.104123)
Supplement: Figure 3—source data 1. [file elife-104123-fig3-data1.pdf]

Detected by blotting with anti-Kbhb

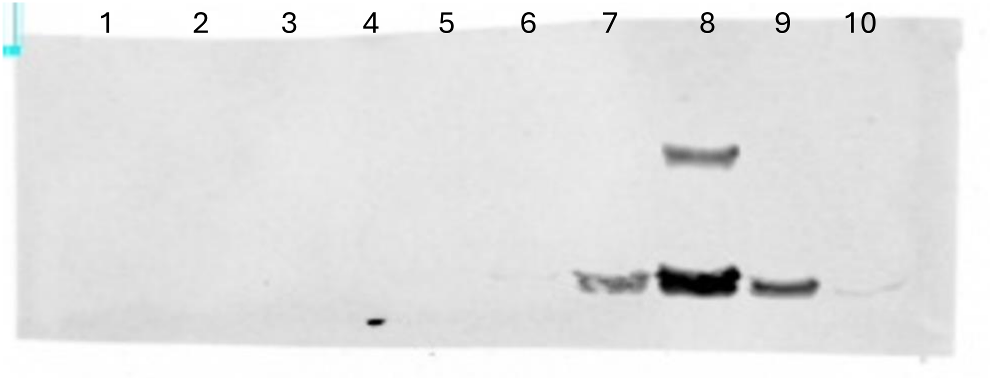

Ponceau staining

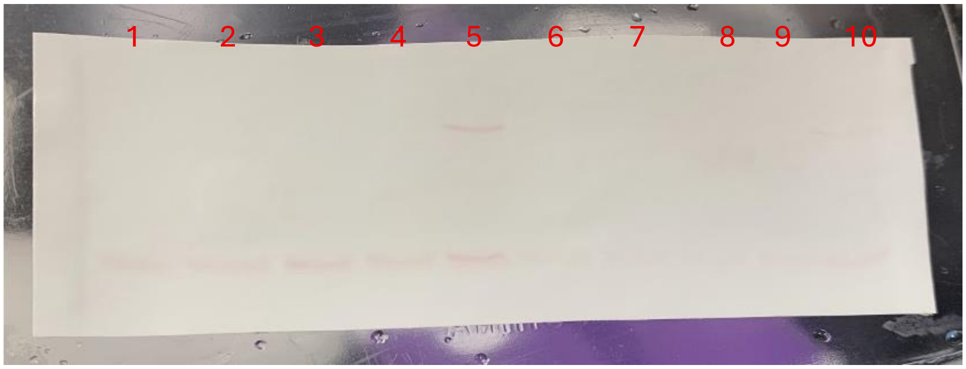

Detected by blotting with anti-Kac

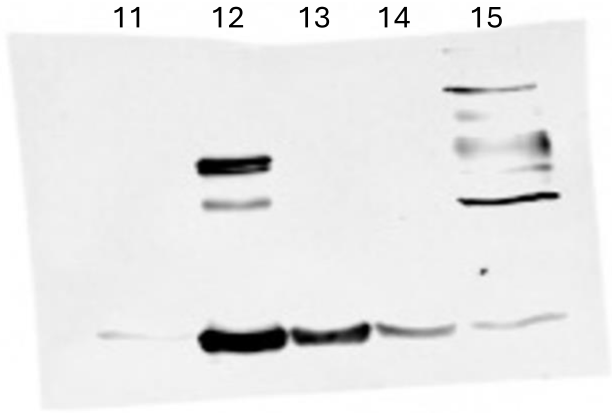

Ponceau staining

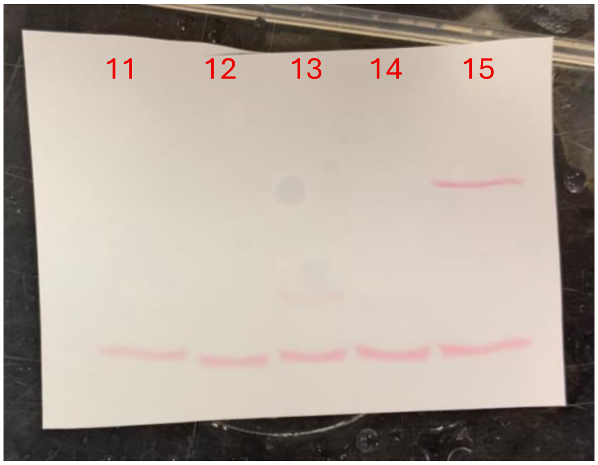

Figure 3, Source Data 1. (A) Original membranes corresponding to Figure 3, panel A. Lanes 1–5 show non–$NaBH_4$-reduced samples from reactions in which histone H3 was incubated with acetoacetyl-CoA in the absence of enzyme or in the presence of p300, PCAF, GCN5, or HBO1. Lanes 6–10 show the corresponding $NaBH_4$-reduced samples from the same HAT reactions. Lanes 11–15 show samples in which histone H3 was incubated with acetyl-CoA in the absence of enzyme or in the presence of p300, PCAF, GCN5, or HBO1.

Detected by blotting with anti-Kbhb

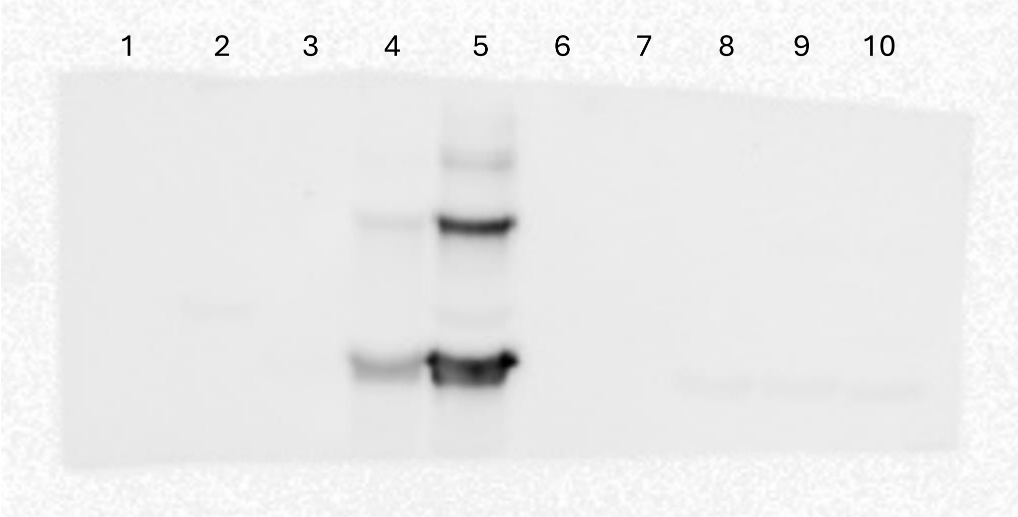

Ponceau staining

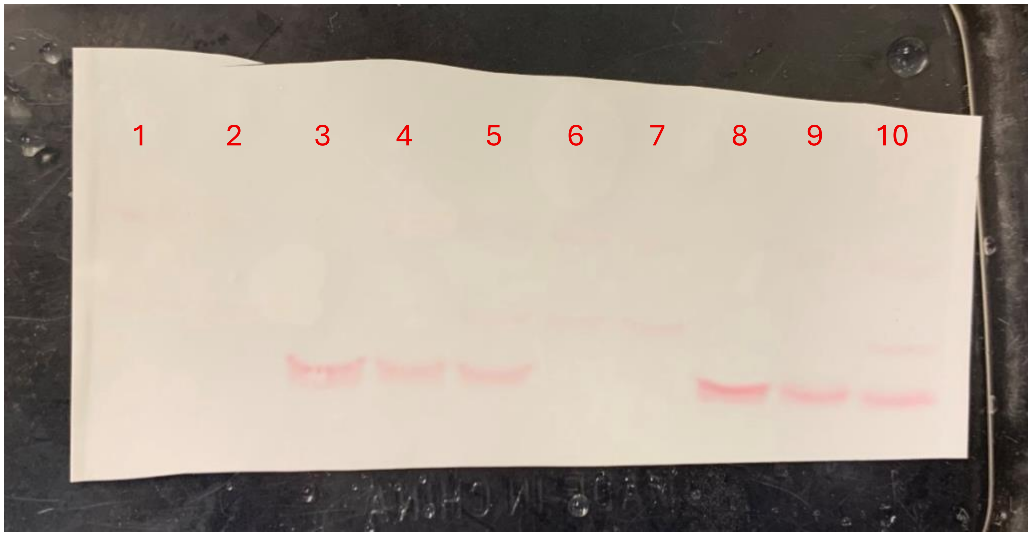

Figure 3, Source Data 1. (B) Original membranes corresponding to Figure 3, panel B. Lanes 1–5 show $NaBH_4$-reduced samples from reactions containing GCN5 alone; acetoacetyl-CoA plus GCN5; histone H3 alone; histone H3 plus acetoacetyl-CoA; histone H3 incubated with acetoacetyl-CoA and GCN5. Lanes 6–10 show the corresponding non–$NaBH_4$-reduced samples under the same conditions.

Detected by blotting with anti-Kbhb

1 2 3 4 5 6 marker

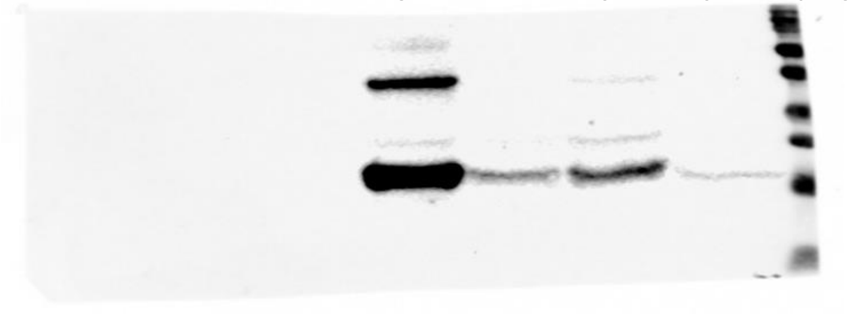

Detected by blotting with anti-Kac

Marker 1 2 3 4 5 6

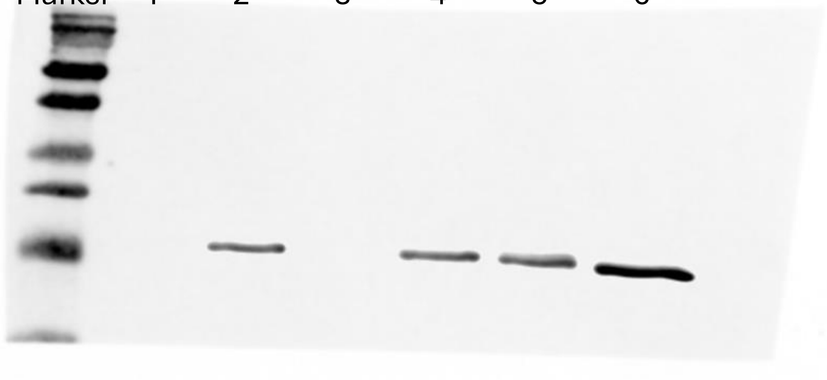

Ponceau staining

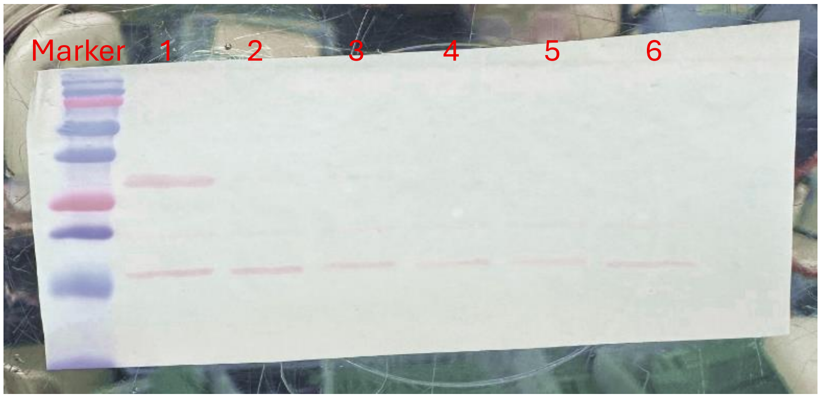

Figure 3, Source Data 1. (C) Original membranes corresponding to Figure 3, panel C. Lanes 1–6 show $NaBH_4$-reduced samples from reactions in which histone H3 and GCN5 were incubated with no added substrate; 10 μM acetyl-CoA; 10 μM acetoacetyl-CoA; 10 μM acetyl-CoA plus 10 μM acetoacetyl-CoA; 10 μM acetyl-CoA plus 50 μM acetoacetyl-CoA; or 50 μM acetyl-CoA plus 10 μM acetoacetyl-CoA.

Detected by blotting with anti-Kbhb

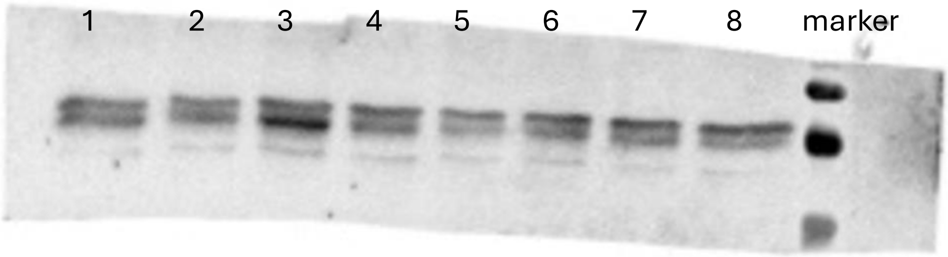

Ponceau staining

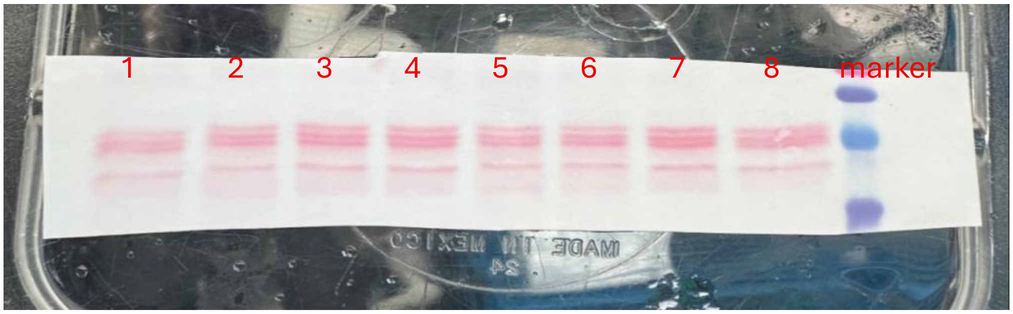

Figure 3, Source Data 1. (E) Original membranes corresponding to Figure 3, panel E. Lanes 1–4 show $NaBH_4$-reduced histone samples from HEK293T cells subjected to HDAC3 overexpression plus acetoacetate treatment; HDAC3 overexpression; acetoacetate treatment; no treatment. Lanes 5–8 show the corresponding non–$NaBH_4$-reduced histone samples under the same conditions.
