# Supplementary material for: Identification of the regulatory elements and protein substrates of lysine acetoacetylation
Source: eLife. 2026 May 14;14:RP104123. doi: 10.7554/eLife.104123 (PMC13175576; doi:10.7554/eLife.104123)
Supplement: Figure 3—figure supplement 1—source data 1. [file elife-104123-fig3-figsupp1-data1.pdf]

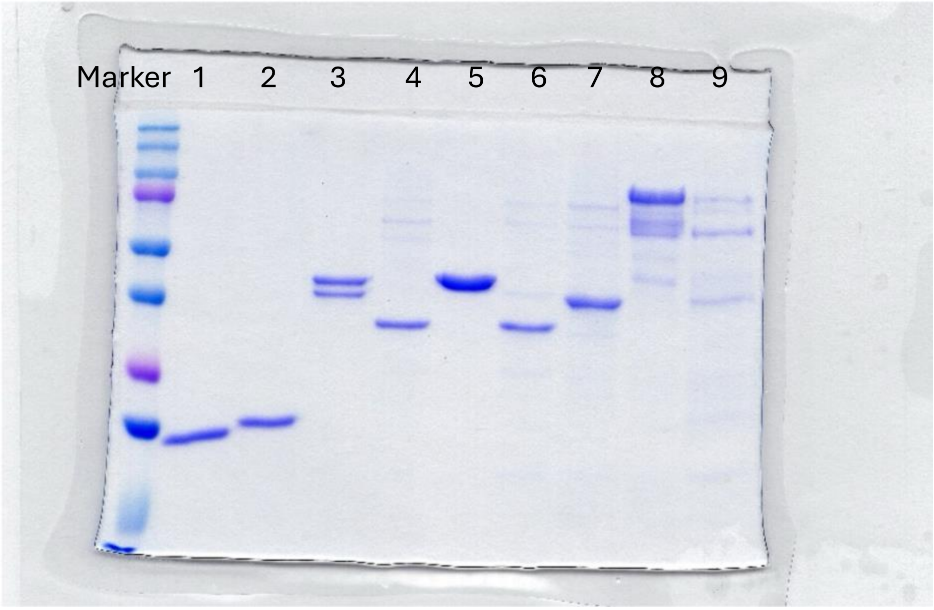

Figure 3-figure supplement 1, Source Data 1. (A) Original SDS-PAGE gel corresponding to Figure 3-figure supplement 1, panel A. Lanes 1–9 display GCN5, PCAF, p300, Tip60, MOF, MOZ, HAT1, MORF, and HBO1, respectively.

Detected by blotting with anti-Kac

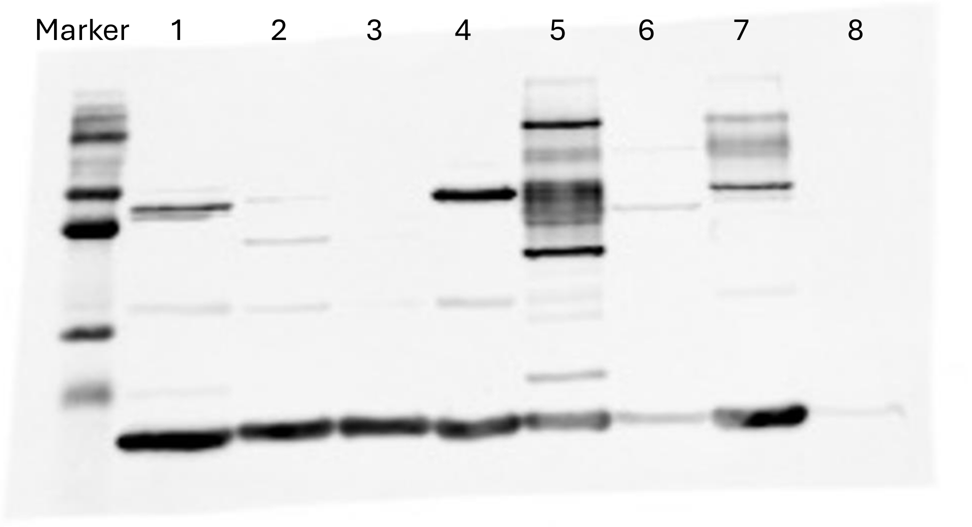

Ponceau staining

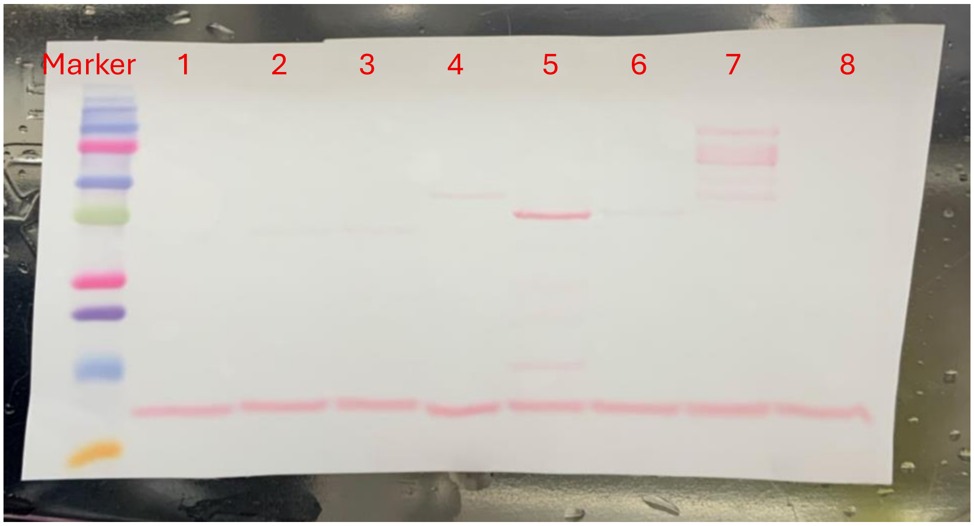

Figure 3-figure supplement 1, Source Data 1. (B) Original membranes corresponding to Figure 3-figure supplement 1, panel B. Lanes 1–8 show histone H4 samples incubated with acetyl-CoA either in the presence of p300, Tip60, MOZ, MOF, HBO1, HAT1, or MORF, or in the absence of enzyme.

Detected by blotting with anti-Kbhb

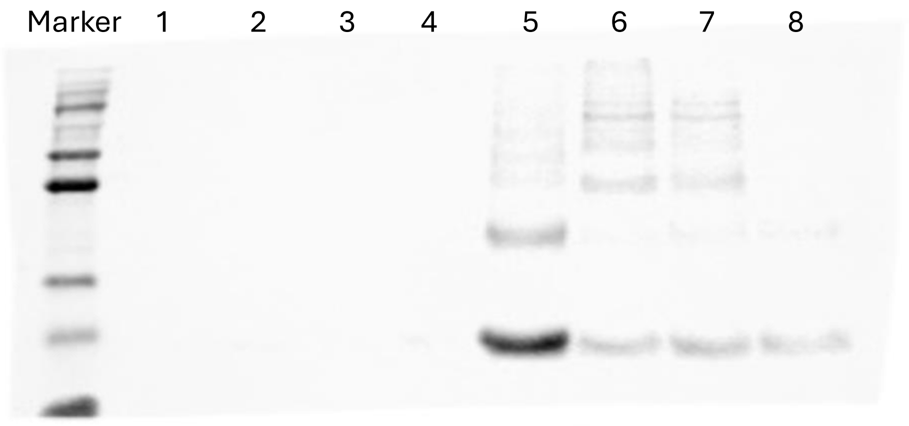

Ponceau staining

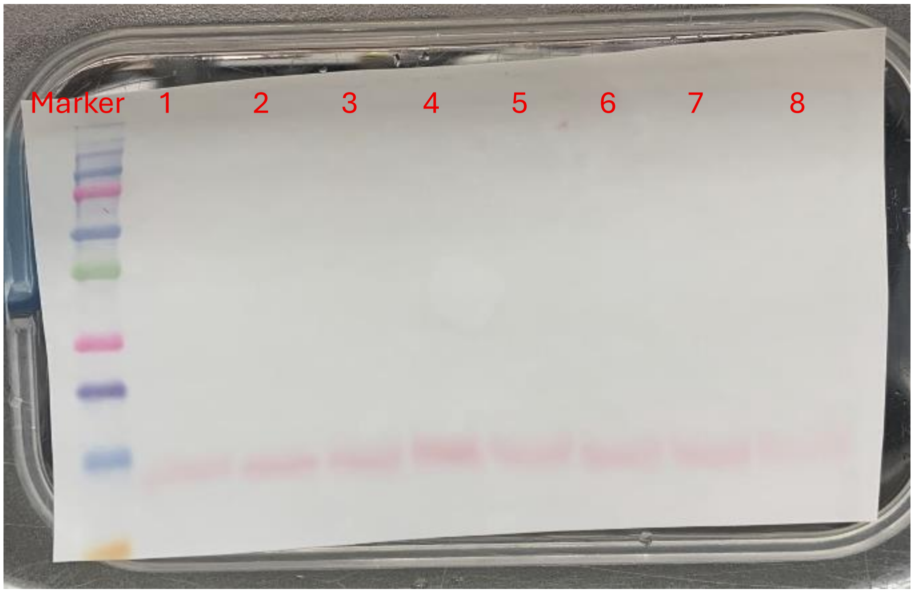

Figure 3-figure supplement 1, Source Data 1. (C) Original membranes corresponding to Figure 3-figure supplement 1, panel C. Lanes 1–4 show non–$NaBH_4$-reduced samples from reactions in which histone H4 was incubated with acetoacetyl-CoA either in the presence of p300, Tip60, or MOZ, or in the absence of enzyme. Lanes 5–8 show the corresponding $NaBH_4$-reduced samples from the same HAT reactions.

Detected by blotting with anti-Kbhb

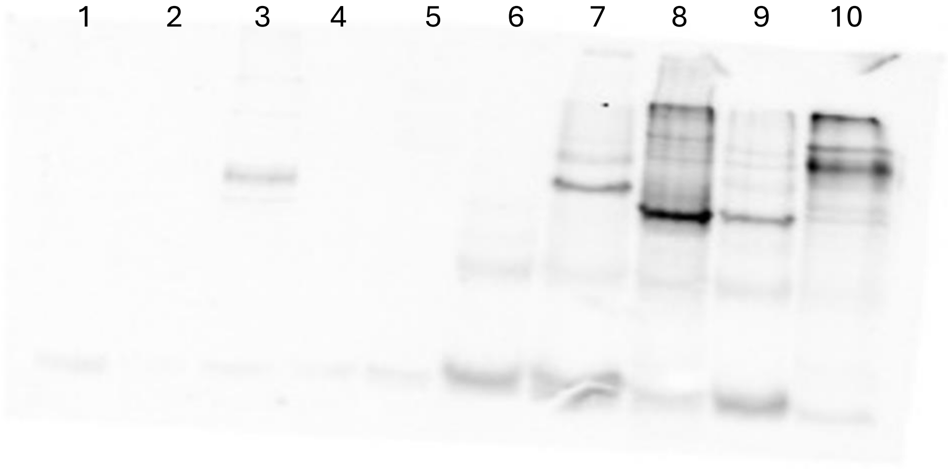

Ponceau staining

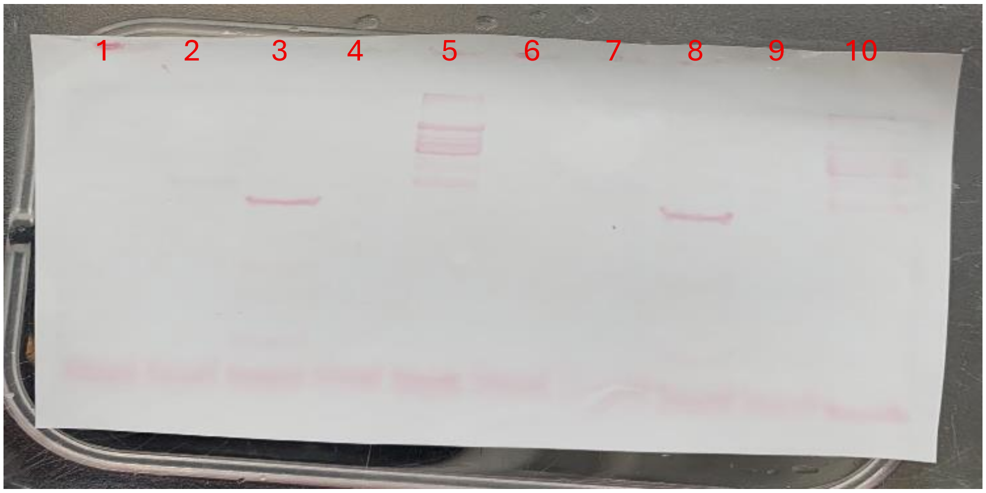

Figure 3-figure supplement 1, Source Data 1. (D) Original membranes corresponding to Figure 3-figure supplement 1, panel D. Lanes 1–5 show non–$NaBH_4$-reduced samples from reactions in which histone H4 was incubated with acetoacetyl-CoA either in the absence of enzyme or in the presence of MOF, HBO1, HAT1, or MORF. Lanes 6–10 show the corresponding $NaBH_4$-reduced samples from the same HAT reactions.

Detected by blotting with anti-Kbhb

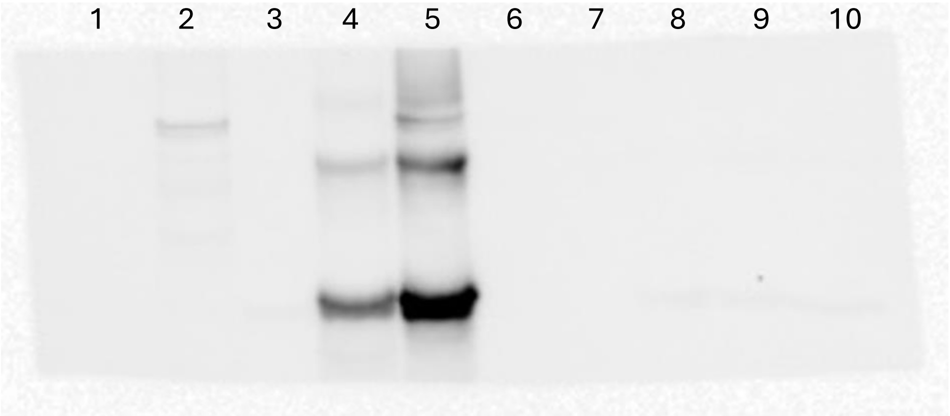

Ponceau staining

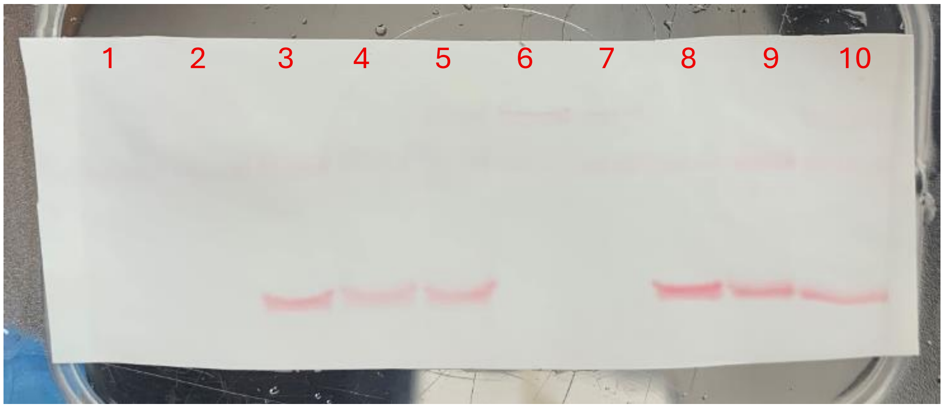

Figure 3-figure supplement 1, Source Data 1. (E) Original membranes corresponding to Figure 3-figure supplement 1, panel E. Lanes 1–5 show $NaBH_4$-reduced samples from reactions containing p300 alone; acetoacetyl-CoA with p300; histone H3 alone; histone H3 with acetoacetyl-CoA; and histone H3 incubated with both acetoacetyl-CoA and p300. Lanes 6–10 show the corresponding non–$NaBH_4$-reduced samples under the same conditions.

Detected by blotting with anti-Kbhb

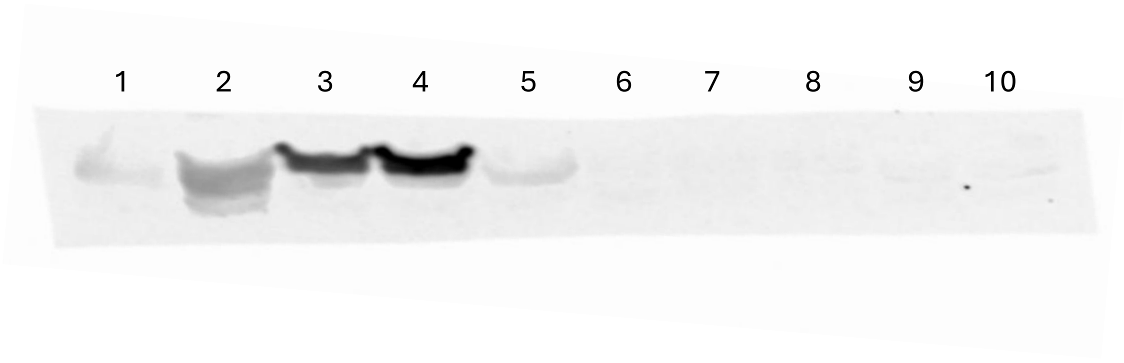

Ponceau staining

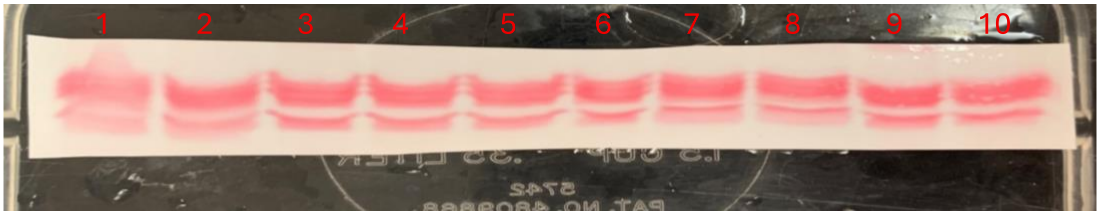

Figure 3-figure supplement 1, Source Data 1. (F) Original membranes corresponding to Figure 3-figure supplement 1, panel F. Lanes 1–5 show $NaBH_4$-reduced samples from reactions in which cellular histone extracts were incubated with acetoacetyl-CoA either in the absence of enzyme or in the presence of p300, GCN5, PCAF, or HAT1. Lanes 6–10 show the corresponding non–$NaBH_4$-reduced samples from the same HAT reactions.

Detected by blotting with anti-Kbhb

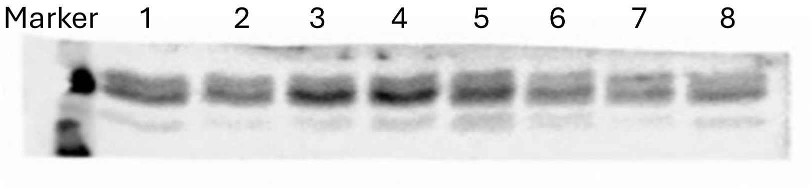

Ponceau staining

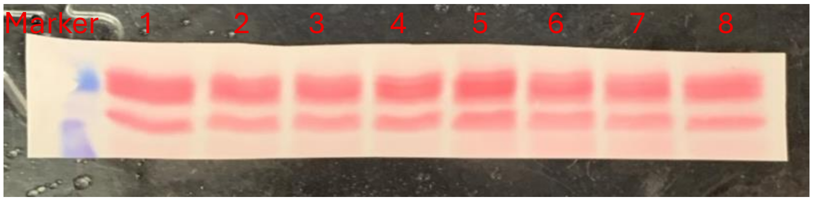

Figure 3-figure supplement 1, Source Data 1. (G) Original membranes corresponding to Figure 3-figure supplement 1, panel G. Lanes 1–4 show $NaBH_4$-reduced histone samples from HEK293T cells under the following conditions: no treatment, p300 overexpression, p300 overexpression with acetoacetate treatment, and acetoacetate treatment. Lanes 5–8 show the corresponding non–$NaBH_4$-reduced histone samples under the same conditions.
