# Supplementary material for: Identification of the regulatory elements and protein substrates of lysine acetoacetylation
Source: eLife. 2026 May 14;14:RP104123. doi: 10.7554/eLife.104123 (PMC13175576; doi:10.7554/eLife.104123)
Supplement: Figure 3—figure supplement 2—source data 1. [file elife-104123-fig3-figsupp2-data1.pdf]

Detected by blotting with anti-Kbhb

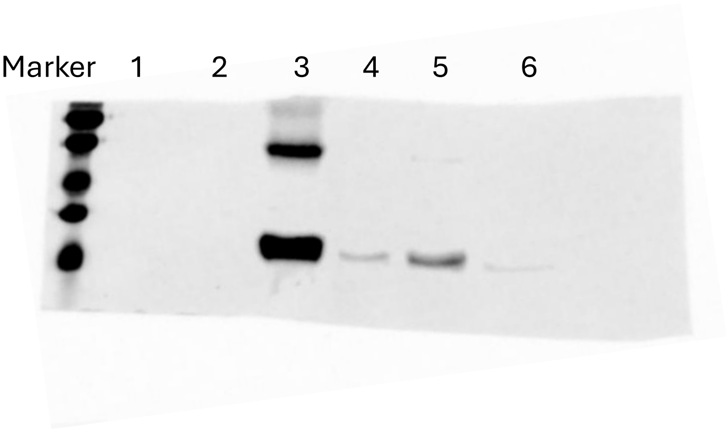

Detected by blotting with anti-Kac

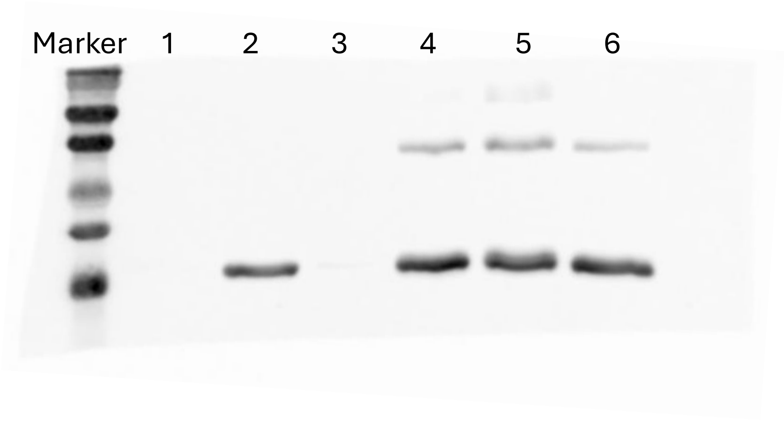

Ponceau staining

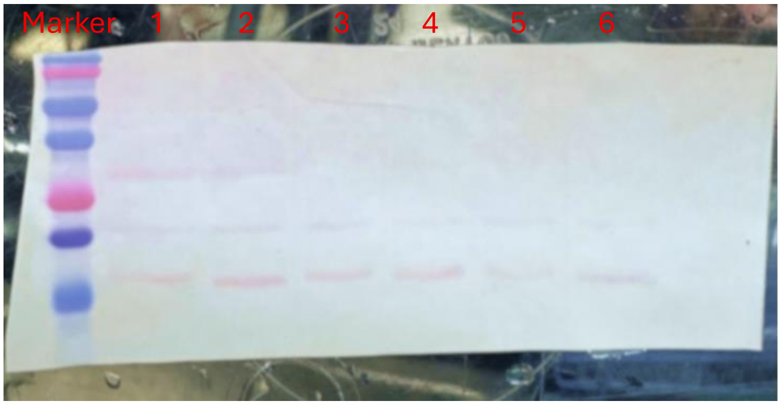

Figure 3-figure supplement 2, Source Data 1. (B) Original membranes corresponding to Figure 3-figure supplement 2, panel B. Lanes 1–6 show $NaBH_4$-reduced samples from reactions in which histone H3 and PCAF were incubated with no added substrate; 10 μM acetyl-CoA; 10 μM acetoacetyl-CoA; 10 μM acetyl-CoA plus 10 μM acetoacetyl-CoA; 10 μM acetyl-CoA plus 50 μM acetoacetyl-CoA; or 50 μM acetyl-CoA plus 10 μM acetoacetyl-CoA.

Detected by blotting with anti-Kbhb

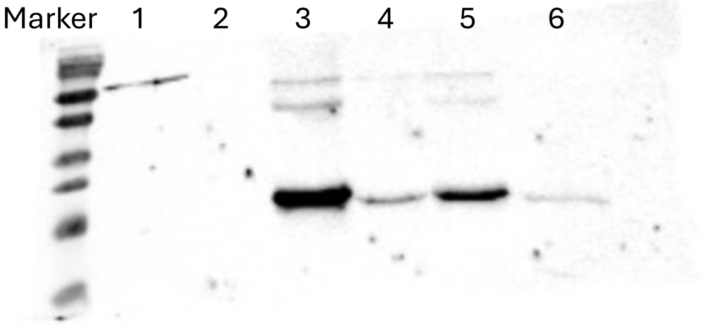

Detected by blotting with anti-Kac

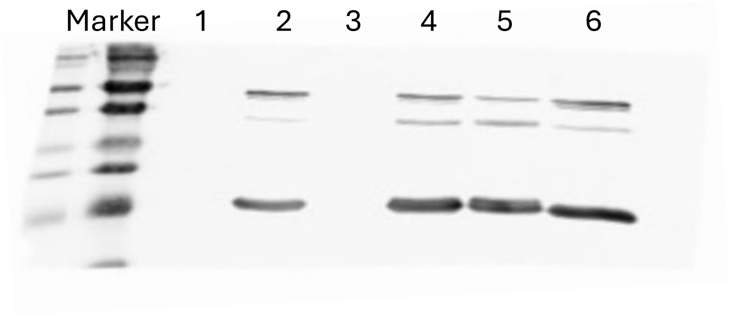

Ponceau staining

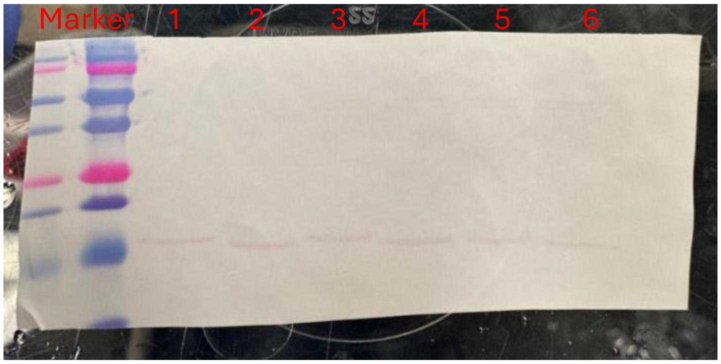

Figure 3-figure supplement 2, Source Data 1. (C) Original membranes corresponding to Figure 3-figure supplement 2, panel C. Lanes 1–6 show $NaBH_4$-reduced samples from reactions in which histone H3 and p300 were incubated with no added substrate; 10 μM acetyl-CoA; 10 μM acetoacetyl-CoA; 10 μM acetyl-CoA plus 10 μM acetoacetyl-CoA; 10 μM acetyl-CoA plus 50 μM acetoacetyl-CoA; or 50 μM acetyl-CoA plus 10 μM acetoacetyl-CoA.

Detected by blotting with anti-Kbhb

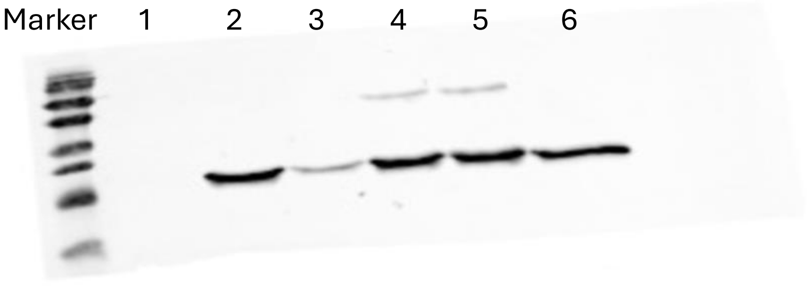

Detected by blotting with anti-Kbhb

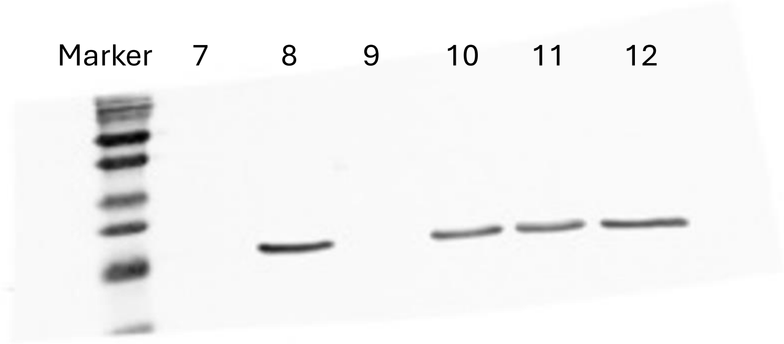

Ponceau staining

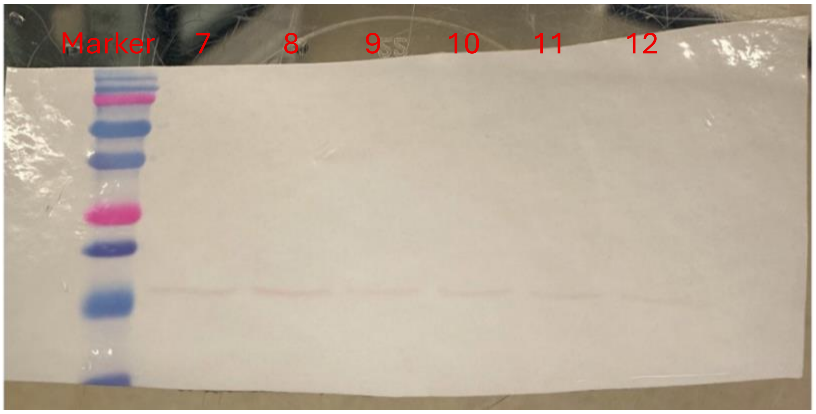

Figure 3-figure supplement 2, Source Data 1. (D) Original membranes corresponding to Figure 3-figure supplement 2, panel D. Lanes 1–6 show $NaBH_4$-reduced samples from reactions in which histone H3 and p300 were incubated with no added substrate; 10 μM β-hydroxybutyryl-CoA; 10 μM acetoacetyl-CoA; 10 μM β-hydroxybutyryl-CoA plus 10 μM acetoacetyl-CoA; 10 μM β-hydroxybutyryl-CoA plus 50 μM acetoacetyl-CoA; or 50 μM β-hydroxybutyryl-CoA plus 10 μM acetoacetyl-CoA. Lanes 7–12 show the corresponding non–$NaBH_4$-reduced samples under the same conditions.
